# Revisiting the role of MicroRNAs in the pathogenesis of idiopathic pulmonary fibrosis

**DOI:** 10.3389/fcell.2024.1470875

**Published:** 2024-10-16

**Authors:** Zhimin Zhou, Yuhong Xie, Qianru Wei, Xinyue Zhang, Zhihao Xu

**Affiliations:** The Fourth Affiliated Hospital of School of Medicine, and International School of Medicine, International Institutes of Medicine, Zhejiang University, Yiwu, China

**Keywords:** IPF, miRNA, TGF-β1/Smad, MAPK, PI3K/AKT

## Abstract

Idiopathic pulmonary fibrosis (IPF) is a prevalent chronic pulmonary fibrosis disease characterized by alveolar epithelial cell damage, fibroblast proliferation and activation, excessive extracellular matrix deposition, and abnormal epithelial-mesenchymal transition (EMT), resulting in tissue remodeling and irreversible structural distortion. The mortality rate of IPF is very high, with a median survival time of 2–3 years after diagnosis. The exact cause of IPF remains unknown, but increasing evidence supports the central role of epigenetic changes, particularly microRNA (miRNA), in IPF. Approximately 10% of miRNAs in IPF lung tissue exhibit differential expression compared to normal lung tissue. Diverse miRNA phenotypes exert either a pro-fibrotic or anti-fibrotic influence on the progression of IPF. In the context of IPF, epigenetic factors such as DNA methylation and long non-coding RNAs (lncRNAs) regulate differentially expressed miRNAs, which in turn modulate various signaling pathways implicated in this process, including transforming growth factor-β1 (TGF-β1)/Smad, mitogen-activated protein kinase (MAPK), and phosphatidylinositol-3-kinase/protein kinase B (PI3K/AKT) pathways. Therefore, this review presents the epidemiology of IPF, discusses the multifaceted regulatory roles of miRNAs in IPF, and explores the impact of miRNAs on IPF through various pathways, particularly the TGF-β1/Smad pathway and its constituent structures. Consequently, we investigate the potential for targeting miRNAs as a treatment for IPF, thereby contributing to advancements in IPF research.

## 1 Introduction

Reports of fibrous lung diseases can be traced back to the 1800s, and the disease of interstitial pulmonary fibrosis was formally defined by *Liebow* and *Carrington* in the late 1960s (Liebow et al., 1965). Of which idiopathic pulmonary fibrosis (IPF) is a fatal, chronic progressive, irreversible interstitial pulmonary fibrosis disease, accounting for 25%–30% of patients with interstitial lung disease. The prevalence of IPF in the Asia-Pacific region ranges from 0.57 to 4.51 per 10,000 people, in Europe it ranges from 0.33 to 2.51 per 10,000 people, and in North America it ranges from 2.40 to 2.98 per 10,000 people (Maher et al., 2021). The mortality rate of IPF is on the rise (Hutchinson et al., 2015), as evidenced by a study from the UK which showed an increase from 0.92 per 100,000 people during the period of 1968–1972 to 5.10 per 100,000 people during the period of 2005–2008 with an annual growth rate of 5% (Navaratnam et al., 2011). Idiopathic means that the exact cause of IPF is unknown, but current research suggests that IPF is the product of complex interactions between host genetic susceptibility and environmental factors. Genetic variants include single nucleotide polymorphisms (SNPs) rs35705950 in the mucin 5B (MUC5B) gene promoter region (Seibold et al., 2011), gene changes related to telomere length such as telomerase reverse transcriptase (TERT), telomerase RNA component (TERC), regulator of telomere length 1 (RTEL1), and poly(A)-specific ribonuclease (PARN) (Armanios et al., 2007), heterozygous mutations in surfactant protein C (SPC) (Nogee et al., 2001), and changes in Toll interaction protein (TOLLIP) (Noth et al., 2013). Epidemiological studies emphasize that smoking, dust and metal exposure, microbial infection, and aspiration of gastric contents may increase the risk of developing interstitial pulmonary fibrosis (Raghu et al., 2011). Aging is also an important risk factor for IPF (Selman and Pardo, 2014). Therefore, this condition is more prevalent in elderly males (Schwartz et al., 1994) and individuals with a history of tobacco use (Baumgartner et al., 1997). Furthermore, there is a growing body of evidence supporting the pivotal role of epigenetic changes in IPF, with epigenetic control of gene expression being recognized as a crucial mechanism for the enduring alterations in gene expression resulting from a combination of genetic, environmental, and other stress-induced phenotypes (Yang and Schwartz, 2011). The median survival time for IPF patients aged 65 and older in the United States after diagnosis is 3.8 years, but it is not uncommon for patients to live longer in real life (Raghu et al., 2014). An open-label clinical trial of pirfenidone demonstrated a median treatment survival time of 77.2 months (Costabel et al., 2017), possibly attributed to significant advancements in drug therapy, as nintedanib and pirfenidone were recommended for IPF treatment in the latest 2022 ATS/ERS/JRS/ALAT guidelines (Raghu et al., 2022). However, lung transplantation remains the only curative intervention (Titman et al., 2009), suitable for only a small number of patients.

**FIGURE 1:**
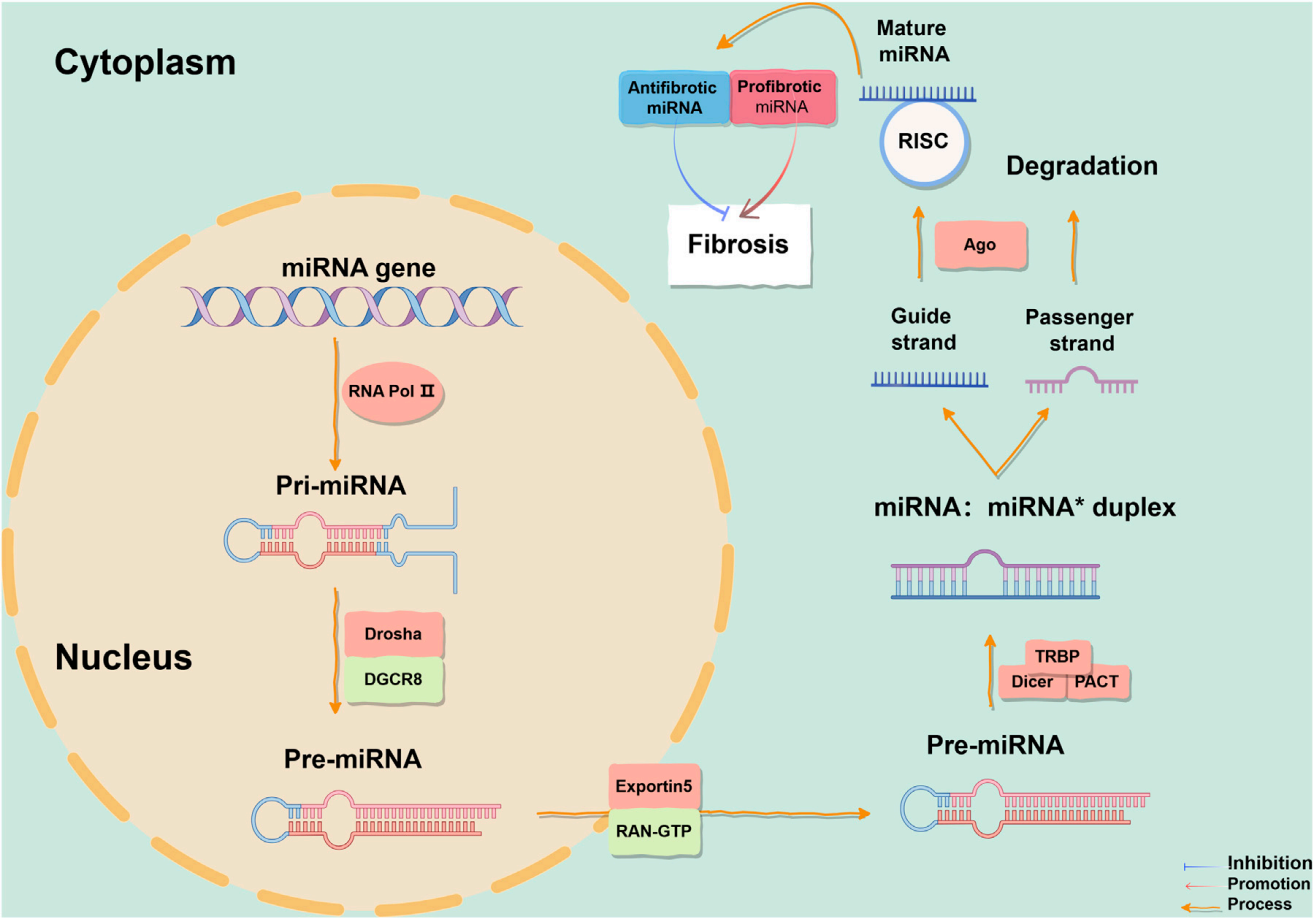
The process of miRNA formation. The biogenesis of miRNAs initiates in the cell nucleus and culminates in the cytoplasm. Mature miRNAs can be categorized into anti-fibrotic miRNAs and pro-fibrotic miRNAs, which modulates fibrogenesis. In this representation, blue denotes inhibitory effects, red arrows signify promoting effects, and orange arrows depict reaction processes. This figure is created by Figdraw. Abbreviations: TGF-β1, transforming growth factor-β1; TβR, TGF-β receptor; DGCR8, DiGeorge Syndrome Critical Region 8; RISC, miRNA-induced silencing complex; Ago, Argonaute; TRBP, transactivation response element RNA-binding protein; PACT, protein activator of protein kinase R.

Non-coding RNA (ncRNA) encompasses microRNA (miRNA) and long non-coding RNA (lncRNA). MiRNAs may indirectly influence gene expression through global effects on methylation and targeting of transcription factors, indicating their effectiveness as the interferent of pulmonary fibrosis (Nana-Sinkam et al., 2009). The discovery of miRNA in the lin-4 gene of *Caenorhabditis elegans* in 1993 marked a significant milestone (Lee et al., 1993), and since then, it has been identified in various organisms, including humans. The human body contains over 1,900 reported miRNAs according to the miRNA database (Kozomara et al., 2019). MiRNAs are single-stranded ncRNAs of length 17–25 nucleotides. Within the nucleus of cells, miRNA genes are transcribed by RNA polymerase II into RNA stem-loop structures of approximately 300–1,000 nucleotides, called primary miRNA (pri-miRNA). The protein DiGeorge Syndrome Critical Region 8 (DGCR8) recognizes the double-stranded RNA structure of pri-miRNA hairpin loops and binds to RNase III enzyme Drosha as part of a complex for processing pri-miRNA into precursor miRNA (pre-miRNA), which is a hairpin-loop structure of 70–90 nucleotides. A pri-miRNA contains several pre-miRNAs. Subsequently, pre-miRNA is transported from the nucleus to the cytoplasm with the assistance of Exportin5 and nuclear protein RAN-GTP, and is cut by Dicer to remove the 3′and 5′ring structures in the cytoplasm, forming mature miRNA double strands (Denli et al., 2004; Lee et al., 2004; Kim, 2004; Ketting et al., 2001). While either strand can function as a functional miRNA, typically only one strand is incorporated into the miRNA-induced silencing complex (miRISC) to target different mRNAs’ 3′untranslated regions (UTRs) by binding to Argonaute proteins, through complementary base pairing to cleave target mRNAs, shorten their poly(A) tails for destabilization, or interfere with mRNA translation for expression control (Rissland et al., 2017; Park and Shin, 2014). The other strand which has a lower expression level, known as the passenger strand and denoted by a star (*), is usually degraded. In some cases, both strands are active and function as functional miRNAs targeting different mRNA populations (Figure 1). There is now substantial evidence supporting the important roles of miRNAs in cellular processes such as apoptosis, proliferation, and differentiation during cell development, and that over 60% of human coding genes are regulated by these molecules. A single miRNA can have multiple targets while multiple miRNAs can regulate the same gene. Investigating the intricate regulatory network of miRNAs can provide insights into eukaryotic genome complexity and related disease mechanisms.

In 2010, it was initially discovered that miRNA expression differed in the lung tissues of patients with IPF and healthy individuals. Interestingly, about 10% of miRNAs showed differential expression in IPF lung tissue compared to normal lung tissue (Pandit et al., 2010; Liu et al., 2010). MiRNAs can be categorized into pro-fibrotic and anti-fibrotic types, each playing distinct roles in the development of IPF. There is evidence that significant alterations in miRNA levels in IPF patients compared to healthy subjects. A microarray analysis of miRNAs revealed that 18 miRNAs were significantly downregulated in IPF lung tissue, including let-7d located in epithelial cells, whose decrease is associated with increased mesenchymal proteins such as N-cadherin, vimentin, and α-smooth muscle actin (α-SMA) (Pandit et al., 2010). Similarly, upregulation of miR-31 expression has been shown to alleviate pulmonary fibrosis in mice (Yang et al., 2012a), suggesting its importance as a regulator in the pathogenesis of IPF. The expression of miR-21 significantly increases in the lungs of both IPF patients and fibrotic mice; its upregulation can be induced by transforming growth factor-β (TGF-β) and promote the epithelial-mesenchymal transition (EMT) process through stimulation of the Smad signal pathway (Liu et al., 2010). Li P. et al. (2014) found abnormal expression of miR-21, miR-101-3p, and miR-155 among patients with impaired forced vital capacity (FVC). The current findings indicate the upregulation of both miR-4443 and miR-4516 in IPF fibroblasts (Sabater et al., 2023). More and more evidence suggests that miRNAs play an important role in the pathogenesis of IPF, and dysregulated miRNAs, together with their targets, form a complex regulatory network related to IPF. Therefore, studying the regulatory mechanisms governing miRNA expression and its role in the development of IPF can help elucidate disease pathogenesis, thereby providing assistance for the discovery of new treatment methods for IPF.

## 2 Regulatory mechanisms of miRNA expression in IPF

**FIGURE 2:**
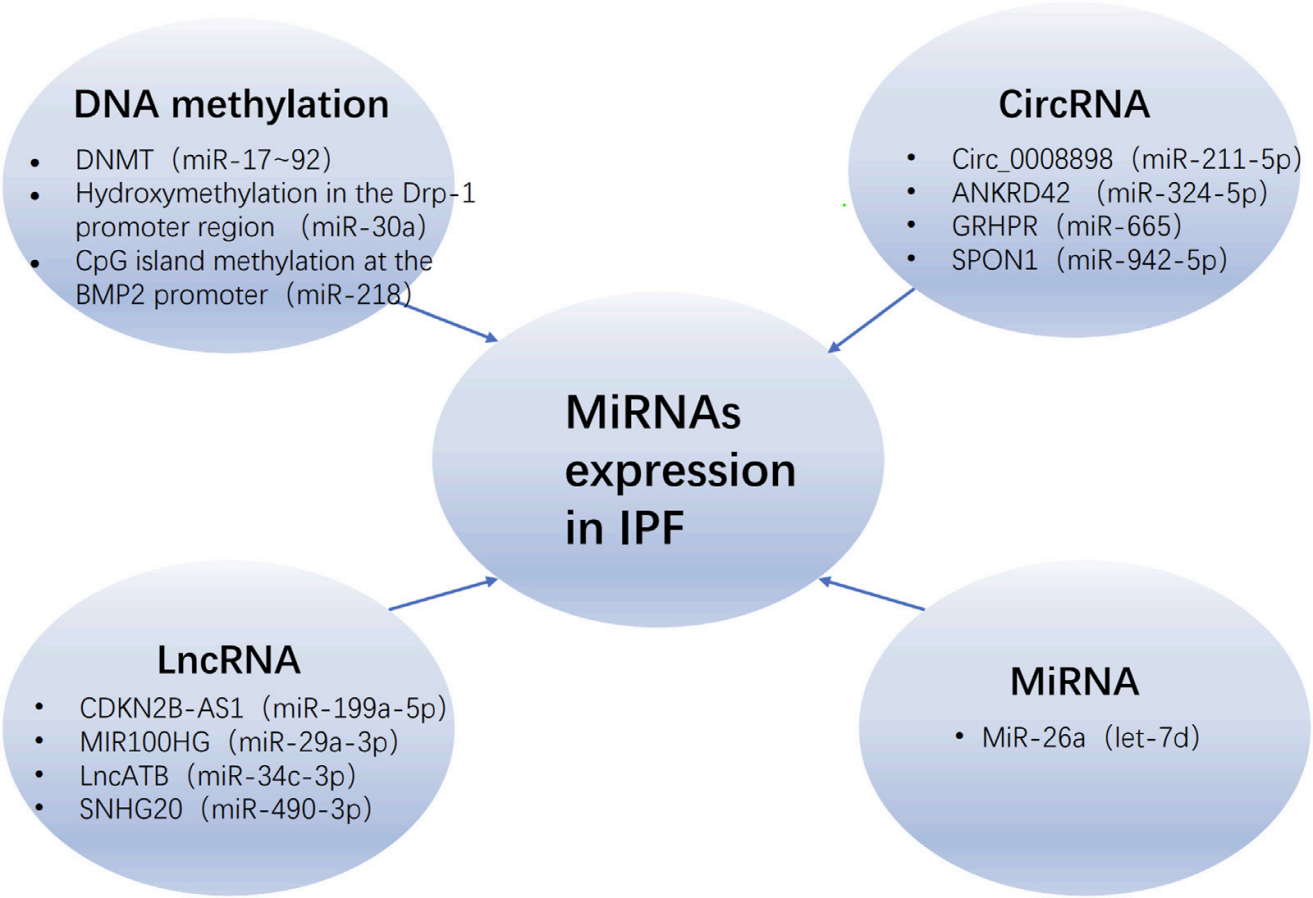
The regulatory of miRNAs expression in IPF. The regulation of miRNAs in IPF is modulated by various epigenetic mechanisms, including DNA methylation, long non-coding RNAs (lncRNAs), circular RNAs (circRNAs), and the miRNAs themselves, with the parentheses denoting those miRNAs that are regulated by these mechanisms. Abbreviations: IPF, idiopathic pulmonary fibrosis; DNMT, DNA methyltransferase; Drp-1, dynamin-related protein-1.

The term “epigenetics” was introduced by scientist C.H. Waddington in 1942 (Richards and Elgin, 2002). Any process that modifies gene function without altering DNA sequence is defined as an epigenetic change, including DNA methylation, histone modification, and dysregulation of non-coding RNA (ncRNA) (Egger et al., 2004). It is widely recognized that epigenetic mechanisms, such as miRNA, play a pivotal role in the pathogenesis and progression of IPF (Zhang et al., 2021). The epigenetic regulation of miRNA in IPF is influenced by a multitude of factors, including DNA methylation, lncRNA, miRNA itself and circular RNA (circRNA) (Figure 2).

MiRNAs can be encoded in the introns, exons, and open reading frames of DNA, and are regulated by DNA promoter elements. If there are DNA methylation sites present, they can be modulated by DNA methyltransferases (DNMTs). In humans, the expression of DNMTs is associated with epigenetic repair of damaged tissues. Bioinformatics analysis suggests that the target genes of miRNAs may play a role in the DNA methylation process through the inhibition of DNMTs (Zhao et al., 2020). Compared to the control group, the expression of miR-17–92 was significantly lower in lung tissue and lung fibroblasts from IPF patients, while the expression of DNMT-1 and the level of DNA methylation at the miR-17-92 promoter were significantly higher. Similarly, treatment with bleomycin (BLM) and 5′-aza-2′-deoxycytidine (a DNMT inhibitor) in mice resulted in enhanced expression of miR-17–92 and weakened expression of fibrotic genes and DNMT-1 (Dakhlallah et al., 2013). These results suggest that there may be an epigenetic feedback loop between miR-17-92 and DNMT-1 in pulmonary fibrosis. Evidence indicates that miR-29c can regulate Fas protein on lung fibroblast surfaces to modulate cell apoptosis sensitivity, as well as directly reduce levels of DNMT-3A and DNMT-3B. However, when mice were treated with the miR-29c inhibitor in a 5′-aza-2′-deoxycytidine environment, the level of Fas did not change (Matsushima and Ishiyama, 2016). Recent experiments have also shown that transfection with miR-29a-3p leads to a decrease in DNMT-3A expression (Cheng et al., 2024). Another molecule, miR-30a, has been shown to attenuate lung fibrosis state in BLM-treated mice by reducing hydroxyproline (HYP), α-SMA, and vimentin expressions while increasing E-cadherin levels, which may be related to its regulation on hydroxymethylation at dynamin-related protein-1 (Drp-1) promoter region, thus inhibiting ten-eleven translocation 1 (TET1, an enzyme converting 5-methylcytosine into 5-hydroxymethylcytosine) expression (Zhang et al., 2017). A recent study (Zhao et al., 2023) has revealed that the overexpression of methyl-CpG binding protein 2 (MeCP2) would exacerbate endothelial-mesenchymal transition (EndMT) and result in increased methylation of the CpG island at the bone morphogenetic protein 2 (BMP2) promoter, thereby promoting fibrosis. The administration of miR-218 can counteract this effect.

LncRNAs, longer than 200 nucleotides, are non-protein-coding transcripts that distinguish them from miRNAs. Most lncRNAs are transcribed by RNA polymerase II and exhibit high tissue and cell specificity due to their less conserved sequences. There is increasing evidence supporting the role of lncRNAs as competitive endogenous RNAs (ceRNAs) in physiological and pathological processes. RT-qPCR analysis reveals that CDKN2B-AS1 is expressed in lung tissue and acts as a ceRNA for miR-199a-5p to mediate Sestrin-2 (SESN-2) activation, promoting cell apoptosis, reducing the expression of fibrosis-related proteins, and thus showing therapeutic potential as an anti-fibrosis target (Yang et al., 2022). MIR100HG regulates the miR-29a-3p/TAB1 (transient activator-binding protein 1) axis to promote fibrosis changes in Type II alveolar epithelial cells (AEC II) (Guan et al., 2022). Similarly, lncATB accelerates EMT progression as a ceRNA for miR-29b-2-5p and miR-34c-3p; co-expression of these two miRNAs using adeno-associated virus (AAV) can better alleviate fibrosis (Xu et al., 2021). Constructing a lncRNA-miRNA-mRNA interaction network can facilitate a more comprehensive understanding of IPF. Bioinformatics analysis of differential expression using GO and KEGG pathways shows that NR_120628/hsa-miR-150-5p/E2F3 may exert a pivotal regulatory role in IPF (Zang et al., 2023). Research has shown that lncRNA MIR155HG is abnormally upregulated in pulmonary fibrosis tissues and normal human primary lung fibroblasts (NHLFs) stimulated by TGF-β1; it binds directly to mir-627 to inhibit its expression, enhancing TGF-β1-induced high mobility group box-1 protein (HMGB1) expression, p65 phosphorylation, NHLFs proliferation, and extracellular matrix (ECM) deposition (Li et al., 2021). LncRNA Snhg6 and PFRL regulate mir-26a to promote BLM-driven mice pulmonary fibrosis process (Deng et al., 2022; Jiang H. et al., 2018). LncNEAT1 is identified as a sponge for miR-9-5p (Zhang et al., 2020) and miR-455-3p (Liu et al., 2021), respectively, which promotes pulmonary fibrosis by regulating the miR-9-5p/TGF-β1/Smad2 and miR-455-3p/Smad3 axes. These findings indicate that the interplay between lncRNA and miRNA plays a crucial role in the pathogenesis of IPF, representing a novel research avenue for elucidating the disease mechanism.

Because one miRNA can have multiple target genes, and multiple miRNAs can regulate the same gene, this complex regulatory network can be a combination of multiple miRNAs to finely regulate the expression of a gene, or it can achieve an impact on the pathology of a disease through the mutual regulation of miRNAs. A study found that increasing miR-26a expression in A549 cells would induce the upregulation of let-7d, but let-7d levels returned to normal after Lin28B treatment (Liang et al., 2016). Interestingly, Lin28B is one of the direct targets of miR-26a, suggesting that miR-26a can regulate the expression of let-7d by targeting Lin28B, thereby promoting the synergistic anti-fibrotic effects of miR-26a and let-7d. Therefore, building a miRNA-miRNA interaction network can enhance our understanding of IPF pathogenesis and merits further exploration and verification.

Sanger et al. were the first to discover circRNA (Sanger et al., 1976), which has a terminal covalent bond that forms a closed loop and is resistant to RNase R degradation, making it more stable than linear RNA. CircRNA originates from the exons or introns of genes and is widely present in eukaryotic organisms, being conserved across different species. In the ceRNA network constructed based on aberrantly expressed circRNAs, miRNAs, and mRNAs, circ_0006916 is upregulated in pulmonary fibrosis. Additionally, miR-199b-5p, miR-296-5p, and miR-708-5p are identified as hub miRNAs that link circRNAs and mRNAs (Wu Q. et al., 2023). These provide important evidence suggesting that abnormal circRNAs and miRNAs may contribute to the development of pulmonary fibrosis. Due to their high cytoplasmic expression and enhanced stability, circRNAs exhibit reduced genetic polymorphisms in miRNA-binding sites compared to other ceRNAs, enabling them to more effectively bind to miRNAs and exert regulatory effects. *In vitro* studies using human lung fibroblasts have confirmed that circ_0001861 modulates the miR-296-5p/BBC3 (BCL2 binding component 3) axis to mitigate pulmonary fibrosis (Wu T. et al., 2023). From a mechanistic standpoint, circ_008898 levels increase in TGF-β1-induced HFL1 cells, leading to competitive binding with miR-211-5p and subsequent upregulation of its target HMGB1 (Zhu M. et al., 2024), thereby exerting an anti-fibrotic effect. In MRC-5 cells, circRNA_ANKRD42 can act as a molecular sponge for miR-324-5p and miR-136-5p, promoting the translation and expression of AJUBA and YAP1 (Xu et al., 2022), and participating in pulmonary fibrosis through close communication with the mechanical rigidity and biochemical signals of the extracellular matrix. Similarly, GRHPR is downregulated in A549 cells induced by TGF-β1 (Wu et al., 2024). Acting as the miR-665 sponge, GRHPR activates the expression of E3 ubiquitin-protein ligase NEDD4-like (NEDD4L), sequentially promoting the ubiquitination of the downstream TGF-βII type receptor (TβRⅡ) and inhibiting aberrant EMT progression. CircSPON1 directly interacts with Smad3 to inhibit fibroblast activation by preventing nuclear translocation (Li et al., 2023). At the same time, circSPON1 binds to miR-942-5p and miR-520f-3p to boost the expression of Smad7. These findings demonstrate that circRNAs can form a series of post-transcriptional regulatory factors by interacting with miRNAs and participate in various signaling pathways involved in pulmonary fibrosis, such as TGF-β1.

## 3 The regulation of IPF by miRNAs involves distinct pathways

### 3.1 TGF-β1/smad pathway

In addition to various types of cells (epithelial cells, endothelial cells, fibroblasts, macrophages, etc.) involved in the pulmonary fibrosis response, several cytokines and their cell signaling pathways also play a dominant role in the fibrosis process. Among them, TGF-β, by recruiting and activating monocytes and fibroblasts, inducing the production of ECM, is an important mediator of fibrosis. Elevated levels of TGF-β are observed in the airway epithelial cells and fibroblasts of IPF patients (Xu et al., 2016). Similarly, in mice, inhibiting TGF-β signal transduction has been shown to delay the progression of pulmonary fibrosis (Li et al., 2011). Therefore, the TGF-β/Smad signaling pathway, as an effective target for anti-fibrosis therapy, is receiving increasing attention.

**FIGURE 3:**
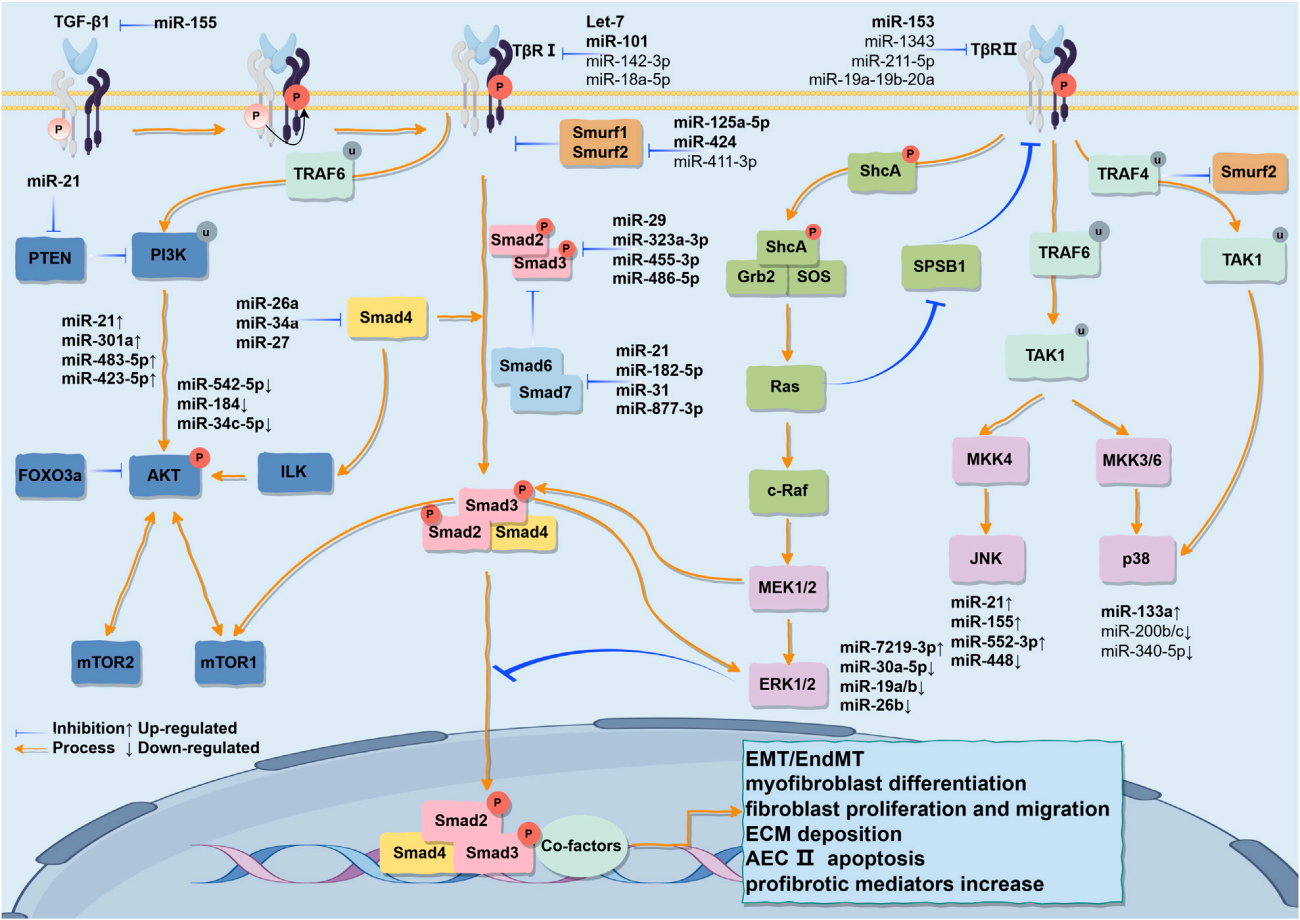
The various signaling pathways activated by TGF-β1 stimulation and their partial cross-talk are outlined. The TGF-β1/Smad pathway is initiated when the TGF-β1 ligand binds to its receptors on the cell membrane, leading to the phosphorylation of Smad2/3 proteins. These phosphorylated Smads subsequently associate with Smad4 to form a trimeric complex that translocates into the nucleus, where it regulates the transcription and translation of fibrosis-related genes, thereby inducing EMT. Concurrently, TGF-β receptors recruit ShcA to initiate downstream signaling mediators Grb2 and SOS, thereby activating the Ras, Raf, MEK, and ERK pathways. Furthermore, TGF-β receptors can simultaneously activate the JNK and PI3K/AKT pathways via TRAF6. Likewise, in the pathogenesis of IPF, miRNAs not only regulate the TGF-β1/Smad signaling pathway but also serve as regulatory factors within this pathway. Additionally, they can interconnect the MAPK and PI3K/AKT pathways, thereby establishing a complex regulatory network that sustains the pathological phenotype. This may open new avenues for utilizing miRNAs as more specific therapeutic agents for IPF. This figure is created by Figdraw.

As a multifunctional cytokine, TGF-β has three isoforms in mammals (TGF-β1, TGF-β2, TGF-β3) with 70%–82% homology at the amino acid level (Meng et al., 2016). It is currently known that TGF-β1 is most closely related to the pathogenesis of IPF. The TβRII on the cell membrane exhibits constitutive activity, and the residues Ser213, Ser409, Tyr259, Tyr336, and Tyr424 are capable of undergoing self-phosphorylation, which is essential for the kinase activity of TβRII (Luo and Lodish, 1997; Lawler et al., 1997). Upon binding to TβRII in a homologous dimeric form (its active form), TGF-β1 phosphorylates the serine/threonine residues in the GS domain (TTSGSGSG) of TβRI, thereby activating TβRI (Wrighton et al., 2009; Wrana et al., 1994). R-Smad (Smad2/3) is regulated by Smad anchor for receptor activation (SARA), Serine/Threonine Kinase Receptor Associated Protein (STRAP), and scaffold protein Axin (Kang et al., 2009), and is recruited and phosphorylated by the activated TβRI at the carboxy-terminal Ser-Ser-X-Ser sequence (called the “SSXS motif”) containing two Ser residues, before forming a heterotrimeric complex with Co-Smad (Smad4) and translocating into the nucleus. There the MH1 domain of R-Smad binds to DNA, while the MH2 domain interacts with other co-factors to regulate fibrosis-promoting genes (Chen and Xu, 2011; Roberts et al., 2001; Massagué, 2012), such as Smad3-dependent regulation of HLF terminal differentiation, stimulating α-SMA transcription (Gu et al., 2007); or activating Smad2 in a time- and concentration-dependent manner to induce AECs to enter the EMT state (Kasai et al., 2005) and EndMT (Jiang Y. et al., 2018). As a part of the negative feedback loop, I-Smads (Smad6 and Smad7) can antagonize signal transduction mediated by R-Smads and Co-Smad, and are considered negative regulators of TGF- β1-mediated fibrosis. Smad7 can compete with and bind to activated TβRI together with Smad2/3, while simultaneously recruiting HECT-domain-containing E3 ligases such as Smurf1, Smurf2, NEDD4-2, or WWP1 to the activated TβRI, resulting in ubiquitination and degradation of the receptors (Huang and Chen, 2012) (Figure 3).

The connection between epigenetics and transcriptional processes is heavily reliant on TGF-β, as evidenced by studies on fibrosis development and glutamate metabolism (Choudhury et al., 2020). Recent research into the regulation of the TGF-β signaling pathway suggests that miRNAs can enhance the biological effects of tissue and temporal control mediated by TGF-β1 in pulmonary fibrosis, namely the modulation of TGF-β1 signaling molecules by pro-fibrotic or anti-fibrotic miRNAs appears to impact the pathogenesis of pulmonary fibrosis (Table 1).

**TABLE 1:** The miRNAs involved in the regulation of the TGF-β1/Smad pathway in IPF.

MiRNA	Expression level in IPF	Experimental model	Validated target	Effect	Function	Reference
miR-21	Upregulated	Animal: BLM fibrosis mice; Cell: MRC-5, IMR-90, HBE	Smad7, Spry1, PTEN	Profibrotic	Induce pulmonary fibroblast activation and collagen deposition, promote TGF-β1 activity	Liu et al. (2010), Yamada et al. (2013); Zhou et al. (2018); Bai et al. (2021)
miR-182-5p	Upregulated	Animal: BLM fibrosis mice; Cell: HELF	Smad7	Profibrotic	Increase fibrin expression by inhibiting Smad7	Chen et al. (2020)
miR-199a-5p	Upregulated	Animal: BLM fibrosis mice; Cell: fibroblasts of IPF, MRC-5, HFL1, A549	CAV1	Profibrotic	Promote proliferation and differentiation	Lino Cardenas et al. (2013)
miR-31	Upregulated	Cell: A549	Smad6	Profibrotic	Induce fibrosis through the TGF-β/Smad2 pathway	Wang et al. (2020a)
miR-144-3p	Upregulated	Cell: Fibroblasts of IPF, MRC-5	RXFP1, TGIF1	Profibrotic	Increase the expression of myofibroblasts	Bahudhanapati et al. (2019), Xu et al. (2015)
miR-125a-5p	Upregulated	Cell: MRC-5, NIH-3T3	Smurf1	Profibrotic	Stimulate fibroblast differentiation	Wang et al. (2020b)
miR-877-3p	Upregulated	Animal: BLM fibrosis mice; Cell: LR-MSCs	Smad7	Profibrotic	Induced differentiation	Wang et al. (2016a)
miR-942-5p, miR-520f-3p	Upregulated	Animal: BLM fibrosis mice; Cell: HFL1	Smad7	Profibrotic	Interference with the expression of Smad7	Li et al. (2023)
miR-410	Upregulated	Animal: BLM fibrosis mice; Cell: HEPF	ADAMTS1	Profibrotic	Stimulate fibroblast proliferation	Liu et al. (2018b)
miR-29	Downregulated	Animal: BLM fibrosis mice; Cell: fibroblasts of IPF, IMR-90	COL1A1, COL4A1, NID1, ITGA11, ADAM12, ADAMTS9, Smad3	Antifibrotic	Inhibit the formation of fibroblasts and collagen synthesis	Khalil et al. (2015), Cushing et al. (2011), Xiao et al. (2012), Tong et al. (2021), Yun and Wang (2024)
miR-200a, miR-200b, miR-200c	Downregulated	Animal: BLM fibrosis mice; Cell: ATⅡ of IPF, MRC-5, RLE-6TN	ZEB1/2	Antifibrotic	Reduce fibrosis	Moimas et al. (2019), Yang et al. (2012b)
miR-26a	Downregulated	Animal: BLM fibrosis mice; Cell: MRC-5, fibroblasts of IPF, HFL-1, A549	Smad4, CCND2, HMGA2	Antifibrotic	Reduce the proliferation and differentiation of fibroblasts	Liang et al. (2014a), Li et al. (2014b), Liang et al. (2014b)
miR-221	Downregulated	Sample: IPF tissues; Animal: BLM fibrosis mice; Cell: A549, HBE	HMGA2	Antifibrotic	Inhibit EMT, alleviate fibrosis	Wang et al. (2016b)
Let-7 family	Downregulated	Sample: IPF tissues; Animal: BLM fibrosis mice; Cell: mouse lung microvascular epithelial line and lung pericyte cell line, A549, RLE-6TN, NHBE	HMGA2, TβRI	Antifibrotic	Reduce cell damage	Pandit et al. (2010), Xie et al. (2020)
miR-101	Downregulated	Sample: IPF tissues; Animal: BLM fibrosis mice; Cell: IMR-90, HFL1, LL29, CCD-8 Lu	FZD4, FZD6, TβRI	Antifibrotic	Inhibit cell proliferation	Huang et al. (2017)
miR-142-3p	Downregulated	Sample: sputum and plasma of patients with IPF	TβRⅠ	Antifibrotic	Reduce the expression of TβRⅠ and pro-fibrotic genes	Guiot et al. (2020)
miR-18a-5p	Downregulated	Cell: HFL1	TβRⅠ	Antifibrotic	Reduce cellular damage and ECM accumulation	Shen et al. (2023)
miR-122-5p	Downregulated	Animal: LPS-lung fibrosis mice; Cell: HPMECs	TβRⅠ	Antifibrotic	Inhibit EndMT and enhance the pulmonary endothelial barrier	Qi et al. (2022)
miR-770-5p	Downregulated	Sample: pneumoconiosis lung tissues; Animal: silica-lung fibrosis mice; Cell: MRC-5	TβRⅠ	Antifibrotic	Inhibit the activation of pulmonary fibroblasts and reduce their migratory ability	Yuan et al. (2021)
miR-490-3p	Downregulated	Animal: Silica-lung fibrosis mice; Cell: MRC-5, NIH/3T3	TβRⅠ	Antifibrotic	Reduce the expression of fibrogenic genes and reverse EMT	Cheng et al. (2021)
miR-153	Downregulated	Animal: BLM fibrosis mice; Cell: MRC-5	TβRⅡ	Antifibrotic	Reduce the pro-fibrotic activity	Liang et al. (2015)
miR-211-5p	Downregulated	Animal: OVA-induced mice; Cell: TC-1	TβRⅡ	Antifibrotic	Inhibit TβR Ⅱ to alleviate pulmonary fibrosis	Zeng et al. (2021)
miR-323a-3p	Downregulated	Sample: IPF tissues; Animal: BLM fibrosis mice; Cell: ATII, 16HBE14o-, human lung fibroblasts	Smad2, TGFA	Antifibrotic	Limit cell apoptosis, repair lung epithelial damage	Ge et al. (2016)
miR-486-5p	Downregulated	Sample:IPF tissues, pneumoconiosis lung tissues; Animal: Silica-lung fibrosis mice, BLM fibrosis mice; Cell: NIH/3T3	Smad2	Antifibrotic	Reduce the distribution and severity of lung lesions	Ji et al. (2015)
miR-448-5p	Downregulated	Animal: OVA-induced mice; Cell: 16HBE	Six1	Antifibrotic	Decrease the proliferation of pulmonary fibroblasts and increase the rate of apoptosis	Yang et al. (2019)
miR-320a-3p	Downregulated	Sample: PF tissues; Cell: A549	STAT3	Antifibrotic	Mitigate EMT	Wang et al. (2021c)
miR-455-3p	Downregulated	Cell: HPAEpiC, BEAS-2B	Smad3	Antifibrotic	Inhibit the ability of cell migration, EMT, and collagen production	Liu et al. (2021)
miR-34a	Downregulated	Animal: Silica-lung fibrosis mice; Cell: A549	Smad4	Antifibrotic	Regulate EMT	Qi et al. (2020)
miR-19a-19b-20a	Downregulated	Animal: BLM fibrosis mice; Cell: Primary lung fibroblasts	TβRⅡ	Antifibrotic	Inhibit fibroblast activation, downregulate pro-fibrotic gene expression	Souma et al. (2018)
miR-411-3p	Downregulated	Animal: Silica-lung fibrosis mice and rats	Smurf2	Antifibrotic	Inhibit the proliferation and migration of pulmonary fibroblasts	Gao et al. (2020)

Abbreviations: IPF, idiopathic pulmonary fibrosis; BLM, bleomycin; HBE, human bronchial epithelial; Spry1, Sprouty RTK Signaling Antagonist 1; HELF, human embryonic lung fibroblast; HFL, human fetal lung fibroblast; CAV1, caveolin-1; RXFP1, Relaxin Family Peptide Receptor 1; TGIF1, TGF-β inducible factor homeobox 1; LR-MSC, lung resident mesenchymal stem cell; HLF, human primary lung fibroblast; HAT2, human type Ⅱ alveolar epithelial cell; COL1, type I collagen; ITGA11, integrin subunit Alpha 11; ADAM12, ADAM metallopeptidase domain 12; ADAMTS9, ADAM, metallopeptidase with thrombospondin type 1 motif 9; ZEB1/2, zinc finger E-box binding homeobox 1/2; CCND2, Cyclin D2; HMGA2, High Mobility Group AT-Hook 2; TβR, TGF-β receptor; LL29, IPF, lung fibroblasts; CCD-8 Lu, normal lung fibroblasts; FZD4, frizzled class receptor 4; HPMEC, human pulmonary microvascular endothelial cells; Six1, SIX, homeobox 1; STAT3, signal transducer and activator of transcription 3; HPAEpiC, human alveolar epithelial cells.

#### 3.1.1 MiRNAs targeting TGF-β1 receptors

The let-7 family is among the earliest miRNAs discovered in humans, comprising let-7a to let-7i. The targets and functions of these miRNAs may exhibit similarities across different animal species. Specifically, let-7a, let-7c, and let-7g target ECM and fibrosis-related genes, rendering them potential candidates for miRNAs with anti-fibrosis contributions. Notably, let-7d is expressed in alveolar epithelial cells and its levels are positively associated with forced vital capacity. TGF-β1 binds to the promoter of let-7d via Smad3 to suppress its expression, resulting in an upregulation of mesenchymal markers such as N-cadherin-2, vimentin, α-SMA, and HMGA2 (Pandit et al., 2010). Furthermore, let-7d can directly target TβRI to drive pulmonary pericardial cell fibrosis in mice (Xie et al., 2020). Using a microRNA microarray analysis of global miRNA expression profiles in 28 IPF patient lung tissues obtained from the Lung Tissue Research Consortium (LTRC), we observed that the expression level of miR-101 was significantly lower. At the same time, this downregulation was also evident in BLM-induced pulmonary fibrosis mice lung tissues. Overexpression of miR-101 in LL29 (IPF lung fibroblasts) resulted in inhibition of α-SMA, COL1A1, and COL3A1 mRNA and protein expression, while anti-miR-101 increased α-SMA and collagen expression in CCD-8 Lu (normal lung fibroblasts). Therefore, it is not surprising that adenovirus-mediated mice lung miR-101 gene transfer alleviated pulmonary fibrosis and improved lung function. Further studies have revealed that miR-101 targets TβRI to inhibit p-Smad2/3, thereby suppressing TGF-β1-induced lung fibroblast activation (Huang et al., 2017). The expressions of miR-142-3p (Guiot et al., 2020), miR-18a-5p (Shen et al., 2023), miR-122-5p (Qi et al., 2022), miR-770-5p (Yuan et al., 2021), and miR-490-3p (Cheng et al., 2021) have been shown in several studies to target TβRⅠ, leading to reduced expression of pro-fibrotic genes and reversal of EMT. It is evident that TβRI plays a crucial role in the TGF-β1/Smad pathway-mediated IPF.

In the current studies, dysregulation of miR-153 has been observed in the IPF mice model. Like other anti-fibrotic miRNAs, the expression of miR-153 in the lungs is repressed by TGF-β1. Increasing miR-153 expression can attenuate TGF-β1’s pro-fibrotic activity, and *vice versa*. It is believed that TβRⅡ is a direct target of miR-153, and the phosphorylation level of downstream Smad2/3 will also be impacted (Liang et al., 2015). Similarly, miR-211-5p alleviates pulmonary fibrosis by inhibiting TβRⅡ (Zeng et al., 2021).

#### 3.1.2 MiRNAs targeting smad proteins

In the lungs, the expression level of miR-29 is inversely associated with pro-fibrotic target gene expression and fibrosis severity. Regarding the relationship between miR-29 and type I collagen (COL1), Khalil et al. found that the mechanism by which miR-29 inhibition IPF fibroblasts and polymerized collagen involves the reduction in the function of protein phosphatase (PP) 2A, which leads to lessening phosphorylation of histone deacetylase 4 (HDAC4) and a reduction in the nuclear translocation of HDAC4. Overexpression of HDAC4 can restore the level of miR-29 and reduce COL1 expression (Khalil et al., 2015). Furthermore, when miR-29 is endogenously knocked out in IMR-90 cells, there is a marked tendency for collagen, MMPs, laminin, and integrin to become less inhibited (Cushing et al., 2011). Similarly, in BLM-induced mice, miR-29 is an upstream target gene of Smad3 and is significantly suppressed by TGF-β1 during fibrosis. Conversely, miR-29 overexpression can negatively regulate TGF-β1 and CTGF expression, as well as the signal transduction of Smad3 (Cushing et al., 2011; Xiao et al., 2012; Tong et al., 2021). In mice with pulmonary fibrosis, tail-intravenous injection of miR-29a agomir would result in a significant reduction in TGF-β1 and CTGF content, the severity of pulmonary fibrosis, as well as the suppression of Smad3 expression in the nucleus (Yun and Wang, 2024). This further proves that overexpression of miR-29a can inhibit the TGF-β1/Smad3 signaling pathway and decrease the degree of pulmonary fibrosis in mice. In other studies, it has been demonstrated that miR-455-3p (Liu et al., 2021) negatively regulates p-Smad3 levels. This miRNA leads to a reduction in fibronectin, collagen IV, and vimentin expression, and restrains EMT and fibrosis induced by TGF-β1.

MiR-26a functions as an endogenous inhibitor of the TGF-β1/Smad signaling pathway, and its downregulation in IPF and pulmonary fibrosis mice models leads to the upregulation of CTGF, COL1A1, and COL3A1. Overexpression of miR-26a can slow down TGF-β1-induced fibrosis in MRC-5 cells and alleviate experimental pulmonary fibrosis in mice. Analysis of promoter sequences using the Genomatix algorithm revealed a binding site for Smad3 upstream of the miR-26a gene, suggesting a regulatory loop between miR-26a and p-Smad3. Through direct targeting of Smad4, miR-26a inhibits the nuclear translocation of p-Smad3, thereby blocking TGF-β1 downstream signal transduction and mitigating pulmonary fibrosis (Liang et al., 2014a). Additionally, miR-26a mediates the TGF-β2-TGF-β1 feedback loop and refrains the TGF-β1-CTGF-collagen pathway and TβRI activation (Li X. et al., 2014). Furthermore, inhibition of miR-26a expression can facilitate the transformation of pulmonary epithelial cells into myofibroblasts both *in vitro* and *in vivo*, that the reason for this may be attributed to the targeting of a crucial factor in the EMT process, HMGA2, by miR-26a (Liang et al., 2014b). An intervention model involving miR-34a was established in A549 cell models stimulated by TGF–β1. It was observed that the expression of miR-34a was downregulated. Upon overexpression of miR-34a, the level of Smad4 mRNA decreased in A549 cells. Therefore, it is hypothesized that miR-34a may partially regulate EMT by targeting Smad4 (Qi et al., 2020).

The researchers have confirmed that miR-31 enhances the formation and translocation of the Smad2/Smad4 dimer, and prevents the dephosphorylation of Smad2 by binding to Smad6 (Wang CJ. et al., 2020).

MiR-21 was the first miRNA investigated in the BLM-induced fibrosis mice model (Liu et al., 2010). Studies have demonstrated that miR-21 is a pro-fibrotic miRNA primarily localized on myofibroblasts in lung fibrotic mice and IPF patients. TGF-β1 can upregulate the expression of miR-21 in primary lung fibroblasts. Administration of the miR-21 inhibitor can prevent the upregulation of vimentin and α-SMA in lung epithelial cells and alleviate EMT (Yamada et al., 2013; Zhou et al., 2018). One potential mechanism by which miR-21 contributes to fibrosis is through its regulation of Smad7 expression and enhancement of p-Smad2 to augment the pro-fibrotic activity of TGF-β1 in fibroblasts (Liu et al., 2010), as recently confirmed in a study (Bai et al., 2021). In IMR-90 cells, Smad3 and Smad complexes are able to bind to the promoter region of miR-21 to promote its expression (Zhou et al., 2018). Analogously, in BLM-induced pulmonary fibrosis mice models and TGF-β1 treated human embryonic lung fibroblasts (HELFs) models, the expression of miR-182-5p was significantly upregulated. Inhibition of miR-182-5p could result in the downregulation of fibronectin, α-SMA, p-Smad2/p-Smad3 expression, and an increase in Smad7 levels. In the meantime, Smad7 is a direct target of miR-182-5p and is negatively regulated by it (Chen et al., 2020). Functional studies using the mice model treated with BLM and lung resident mesenchymal stem cells (LR-MSCs) revealed that decreased expression of miR-877-3p could suppress LR-MSCs myofibroblast differentiation and attenuate pulmonary fibrosis by targeting Smad7 (Wang C. et al., 2016). In fibroblasts, miR-942-5p and miR-520f-3p are capable of modulating Smad7 mRNA and disrupting Smad7 expression (Li et al., 2023).

#### 3.1.3 MiRNAs targeting additional cellular components involved in the TGF-β1/smad signaling pathway

In MRC-5 cells, elevated levels of miR-125a-5p resulted in the upregulation of α-SMA and Smad1, as well as the downregulation of Smurf1 (Wang D. et al., 2020), indicating that miR-125a-5p may target the TGF-β1/Smad1 signaling pathway and facilitate fibroblast differentiation into myofibroblasts.

Meanwhile, upregulation of miR-411-3p can inhibit the proliferation and migration of pulmonary fibroblasts, reduce the ubiquitination and degradation of Smad7, and achieve the goal of blocking the TGF-β1/Smad signal transduction pathway (Gao et al., 2020). Moreover, the miRNA microarray and real-time fluorescence quantitative PCR analysis were utilized in human lung epithelial cell EMT models, revealing the upregulation of miR-424 and its ability to increase α-SMA levels. Subsequent experiments demonstrated that miR-424 reduced the protein expression of Smurf2 (Xiao et al., 2015). A recent study showed a 2.6-fold increase in miR-424 in TGF-β1-induced HLF compared to non-fibrotic lung tissue, as well as a 1.7-fold increase in IPF patient lung tissue. Treatment with the Smad3 inhibitor SIS3 blocked the upregulation of miRNA, while treatment with a miR-424 inhibitor resulted in reduced phosphorylation of Smad3. The luciferase reporter gene assay revealed that the TGF-β1 signal transduction inhibitor protein Slit2 was targeted by miR-424 which leads to the downregulation of Slit2 and induction of α-SMA and connective tissue growth factor (CTGF) expression (Huang et al., 2020). These findings suggest that miR-424 is a pro-fibrotic miRNA that inhibits the expression of anti-fibrotic factor Slit2 through reliance on Smad3, serving as a positive feedback regulator of the TGF-β1 signaling pathway. Interestingly, during the process of repairing damage and maintaining lung homeostasis, the upregulation of miR-424 and miR-503 inhibits the expression of serum/glucocorticoid receptor kinase 1 (SGK1), thereby suppressing glucocorticoid signal transduction and inducing AEC II differentiation into AEC I (Castaldi et al., 2020). Therefore, miR-424 may impact the lung environment through multiple pathways, and the ultimate function of the lung may be influenced by various factors.

#### 3.1.4 MiRNAs exhibiting a dual nature in pulmonary fibrosis

In an array analysis of human lung fibroblasts exposed to reactive oxygen species (ROS), miR-9-5p was found to inhibit the phosphorylation of Smad2 and activation of Smad4 by targeting the inhibition of TβRII and NADPH oxidase 4 (NOX4), resulting in delayed nuclear translocation of Smad2/3. This led to a reduction in the phenotypic transition of interstitial cells towards myofibroblasts. The inhibitory effect of miR-9-5p on TGF-β1-induced phosphorylation of Smad2 was weakened when exogenous TβRII was introduced. Similarly, miR-9-5p demonstrated significant anti-fibrotic effects in a BLM-induced mouse pulmonary fibrosis model (Fierro-Fernández et al., 2015). However, in the BLM-induced IPF mouse model, bioinformatics prediction and dual luciferase reporter gene assay revealed that miR-9 inhibited the activation of the TGF-β1/Smad3 pathway by targeting anoctamin-1 (ANO-1), thereby exacerbating the inflammatory response in IPF, promoting the proliferation of pulmonary fibroblasts and inhibiting their apoptosis ability (Dai et al., 2019). Furthermore, it was observed that the expression level of miR-9-5p was upregulated in the lung tissues of IPF patients (Fierro-Fernández et al., 2015). In addition, a study found that miR-590 inhibited the signaling pathway by directly suppressing the TGF-β1 and TGF-β receptor, as well as downregulating the expression of Smad3 (Dirol et al., 2022). However, the plasma level of miR-590 in IPF patients was significantly higher than that of the control group. One potential explanation for this disparity is that during the inflammatory process, signal transducer and activator of transcription 3 (STAT3) can be activated as a target of miR-590 to activate STAT5 which in turn increases the expression of miR-590 (Cocolakis et al., 2008). Therefore, we assume that this may be due to a self-regulatory feedback loop between TGF-β1 and miR-9-5p and miR-590, whereby when the anti-fibrotic function is produced, the other end of the balance reaction triggers a pro-fibrotic function to limit it. Feedback regulation is an important aspect of molecular cascades. It has been reported that miR-155 is transcribed from an exon of a non-coding RNA located on chromosome 21. In endothelial cells, miR-155 exerts negative regulation on its target src homology 2-containing inositol phosphatase-1 (SHIP-1), resulting in significant enhancement of fibrosis and EndMT. Mice with a knockout of the miR-155 gene and endothelial cells transfected with miR-155 inhibitors showed notable reductions in p-Akt, p-Smad2, and p-Smad3 activation, indicating potential involvement of the PI3K/AKT and TGF-β1/Smad signaling pathways (Tang et al., 2020). In a rat model of BLM-induced pulmonary fibrosis, downregulation of miR-155-5p was correlated with significant decreases in TGF-β1 levels as well as reduced HYP and CTGF levels (Adel et al., 2024). Conversely, recent research demonstrated that miR-155 was downregulated in human pulmonary fibroblast cells treated with TGF-β1; furthermore, overexpression of miR-155 mitigated the stimulatory effects of TGF-β1 on fibroblast proliferation, migration, and collagen synthesis. Further investigation revealed that the suppression of miR-155 led to reduced Smad1 gene expression (Chi et al., 2019), thereby uncovering an indirect interaction between miR-155-Smad and TGF-β1 signal transduction. In HFL, the expression of miR-133a is significantly increased in a time- and concentration-dependent manner by the action of TGF-β1. Surprisingly, quantitative proteomic analysis showed that miR-133a targets multiple components of the TGF-β1-induced fibrotic pathway (TβRI, CTGF, and COL1A1) to inhibit myofibroblast differentiation. Moreover, transferring the miR-133 gene into lung tissue can ameliorate BLM-induced pulmonary fibrosis in mice (Wei et al., 2019). Homoplastically, the level of miR-1343 in A549 cells significantly increased following exposure to TGF-β1. Simultaneously, miR-1343 directly targeted the 3′-UTR of TβRⅠ and TβRⅡ, leading to a significant reduction in the level of p-Smad2/3, the effector molecule of TGF-β1. This inhibition resulted in the suppression of EMT and a decrease in fibrosis markers (Stolzenburg et al., 2016). In a separate study, fluorescein enzyme reporter and Western blotting were utilized to demonstrate the binding of miR-27b to the 3′UTR of TβRⅠ and Smad2. Overexpression of miR-27b in lung fibrosis mice and LL29 cells was found to suppress the expression of COL1A1, COL3A1, COL4A1, and α-SMA induced by TGF-β1, thereby reducing cellular contractility (Zeng et al., 2017). Similar evidence has been reported in other studies where miR-27a-3p targets α-SMA, Smad2, and Smad4 to inhibit the differentiation of lung fibroblasts into myofibroblasts. However, the upregulation of miR-27a-3p in TGF-β1-treated human lung fibroblasts is dependent on Smad2/3 (Cui et al., 2016). Xu et al. (2019) discovered a significant increase in miR-145 expression in MRC-5 cells following stimulation. Subsequently, miR-145 suppressed the TGF-β1/Smad pathway, which promotes fibrosis, by targeting a key factor, Smad3. These findings suggest that the presence of one or more positive feedback loops in the fibrosis pathway, and miRNAs may serve as negative feedback regulators of the TGF-β1-induced fibrosis pathway. Modulating their expression could offer a novel therapeutic approach for preventing or partially reversing the progression of IPF (Table 2).

**TABLE 2:** MicroRNAs exhibiting dual roles in pulmonary fibrosis.

MiRNA	Expression level in IPF	Experimental model	Validated target	Effect	Function	Reference
miR-9	Up-regulated	Animal: BLM fibrosis mice	ANO1	Profibrotic	Exacerbate the inflammatory response, promote the proliferation of pulmonary fibroblasts and inhibit their apoptosis ability	Dai et al. (2019)
	Down-regulated	Sample: IPF tissues; Animal: BLM fibrosis mice; Cell: HFL-1, MC	NOX4, TβRⅡ	Antifibrotic	Alleviate the transformation of mesothelial cells into myofibroblasts	Fierro-Fernández et al. (2015)
miR-590	Up-regulated	Cell: HC11	STAT3	Profibrotic	Stimulate an inflammatory response	Dirol et al. (2022), Cocolakis et al. (2008)
	Down-regulated	Sample: blood samples of IPF patients; Cell: SW480	Smad3	Antifibrotic	Inhibit the TGF-β1/Smad pathway to alleviate fibrosis	Dirol et al. (2022)
miR-155	Up-regulated	Animal: BLM fibrosis mice and rats; Cell: MLEC, HUVEC	SHIP-1, IL-1ß, TNF-α, TGF-β1	Profibrotic	Enhanced fibrosis and EndMT	Tang et al. (2020), Adel et al. (2024)
	Down-regulated	Cell: HFL1	Smad1	Antifibrotic	Reduce the proliferation, migration, and collagen synthesis of fibroblasts	Chi et al. (2019)
miR-133a	Up-regulated	Cell: HFL		Profibrotic		Wei et al. (2019)
	Down-regulated	Animal: BLM fibrosis mice; Cell: HFL, 16HBE, NIH3T3	TβRI, CTGF, COL1A1	Antifibrotic	Resist fibrosis through the p38 MAPK pathway	Wei et al. (2019)
miR-1343	Up-regulated	Cell: A549		Profibrotic		Stolzenburg et al. (2016)
	Down-regulated	Cell: A549, 16HBE14o-	TβRⅠ, TβRⅡ, ELMO2	Antifibrotic	Diminish the levels of fibrosis biomarkers and suppress EMT	Stolzenburg et al. (2016)
miR-27	Up-regulated	Cell: MRC-5		Profibrotic		Cui et al. (2016)
	Down-regulated	Animal: BLM fibrosis mice; Cell: LL29, mice fibroblast cells and AEC Ⅱ, MRC-5	α-SMA, TβRⅠ, Smad2, Smad4	Antifibrotic	Inhibit the transdifferentiation of pulmonary fibroblasts	Zeng et al. (2017), Cui et al. (2016)
miR-145	Up-regulated	Cell: MRC-5		Profibrotic		Xu et al. (2019)
	Down-regulated	Cell: MRC-5	Smad3	Antifibrotic	Relieve EMT	Xu et al. (2019)
miR-424	Up-regulated	Cell: A549, HLF, HAT2	Smurf2, Slit2	Profibrotic	Stimulate myofibroblast differentiation and enhance EMT	Xiao et al. (2015), Huang et al. (2020)
	Down-regulated	Cell: human AT2	SGK1	Antifibrotic	Suppression of SGK1 expression facilitates the transdifferentiation of AEC II into AEC I	Castaldi et al. (2020)

Abbreviations: ANO1, Anoctamin 1; MC, mesothelial cell; NOX4, NADPH, Oxidase 4; HC-11, HC11 mammary epithelium; MLEC, mouse lymphatic endothelial cells; HUVEC, human umbilical vein endothelial cells; SHIP-1, inositol polyphosphate-5-phosphatase D; CTGF, connective tissue growth factor; ELMO2, engulfment and cell motility 2; LPS, lipopolysaccharide; SGK1, serum/glucocorticoid receptor kinase 1.

### 3.2 Other pathways that are interfered with by TGF-β1/smad crosstalk and their related miRNAs

TGF-β1 not only directly activates Smad receptors to mediate cell signaling, but also induces the activation of other signal molecules in a cell-type-specific manner, such as mitogen-activated protein kinase (MAPK) and PI3K/Akt (Zhang, 2009), which significantly enhances the TGF-β1 response. In various physiological and pathological conditions, different kinases or signaling pathways modulate the Smad pathway to regulate protein expression. These pathways can facilitate cellular fibrotic changes including cytoskeletal reorganization and suppression of epithelial markers, while also interfacing with genomic signals through regulation and activation of different miRNAs.

#### 3.2.1 MAPK pathway

The MAPKs are composed of three distinct cascades, namely the c-Jun N-terminal kinase (JNK), p38, and extracellular signal-regulated kinase (ERK) pathways. These cascades have the ability to phosphorylate residues within the Smad2 and Smad3 binding domain, thereby modulating Smad3-dependent gene transcription (Kamato et al., 2013). They interact with the TGF-β1/Smad signaling network at various levels and play a crucial role in most cellular processes. Their involvement in IPF has been gradually elucidated.

The activation of the ERK/MAPK pathway by TGF-β1 initiates with ShcA. TGF-β1 phosphorylates the SH2 and PTB domains of TβRI with tyrosine, thereby activating it as a tyrosine receptor kinase. This leads to the recruitment of p52 ShcA to TβRI and concurrent induction of serine and threonine residue phosphorylation in ShcA (Lee et al., 2007). Additionally, due to Src’s ability to phosphorylate Tyr284 of TβRII, ShcA is also able to bind to TβRII (Galliher and Schiemann, 2007). Tyrosine phosphorylated ShcA initiates the binding sites for downstream signal transducers Grb2 and SOS, leading to the activation of Ras GTPase, subsequently triggering the activation of c-Raf, MAPK kinase 1/2 (MEK1/2), and ERK1/2 (Lee et al., 2007). MEK1 activation can stimulate Smad3 transcription, thereby enhancing TGF-β1 signal transduction in epithelial cells and smooth muscle cells (Ross et al., 2007). Meanwhile, the TGF-β1/Smad3 signal can enhance the phosphorylation level of ERK, consequently promoting TGF-β1-induced collagen contraction and migration (Kadoya et al., 2019).

TGF-β1 induces the activation of JNK and p38 in a Smad-independent manner. The TGF-β receptor complex interacts with tumor necrosis factor receptor-associated factor 6 (TRAF6), an E3 ubiquitin ligase, at a conserved consensus site (basic residue-X-P-X-E-X-X-aromatic/acidic residue) through ubiquitination-induced activation of TRAF6. The activation of TRAF6 leads to the activation of MAP kinase kinase kinase (TGF-beta activated kinase 1, also known as MAP3K7, TAK1) through Lys63-linked polyubiquitination (Sorrentino et al., 2008; Yamashita et al., 2008). TAK1 serves as a substrate for MAP kinase kinases (MKKs) and is responsible for activating MKKs. Specifically, phosphorylation of MKK4 leads to JNK activation, while phosphorylation of MKK3/6 results in p38 MAPK activation (Lin et al., 1995). TRAF4 is another E3 ligase capable of inducing the polyubiquitination of TAK1 at Lys63 linkage, thereby directly facilitating the activation of p38 MAPK (Zhang et al., 2013). In comparison to TRAF6, TRAF4 is also capable of stabilizing TGF-β receptor levels and enhancing Smad signaling by inhibiting Smurf2-mediated TβRI degradation (Sorrentino et al., 2008) (Figure 3).

MiRNAs can also facilitate the progression of IPF through the MAPK pathway. Mir-21 is extensively recognized as a key miRNA in the promotion of fibrosis, demonstrating involvement not only in the TGF-β1/Smad pathway but also in the MAPK signaling cascade, where it significantly enhances the phosphorylation levels of ERK, JNK, and p38 (Wang et al., 2018). The previously mentioned miR-155 was further validated in another study as a promoter of fibrosis development (Sun et al., 2021). The application of miR-155 antagomir significantly attenuated the histological alterations and HYP levels induced by BLM, while also silencing MAP3K7 binding protein 2 (TAB2). High-throughput sequencing results indicate that miR-7219-3p is markedly elevated in silicosis mice and facilitates the transdifferentiation of fibroblasts into myofibroblasts. As its target, Spouty1 (SPRY1) functions as a negative regulator of the Ras/ERK/MAPK signaling pathway, and its knockdown significantly enhances cell proliferation and migration (Niu et al., 2022). Another analogous study identified through high-throughput sequencing revealed that miR-552-3p and its predicted target gene Caveolin 1 (CAV1) are associated with pulmonary fibrosis. In bronchoalveolar lavage fluid (BALF), as well as in murine and cellular experiments, the levels of miR-552-3p were found to be elevated, concomitantly activating the associated MAPK signaling pathway (Li M. et al., 2024). In RLE-6TN cells, the combined action of lipopolysaccharide (LPS) and TGF-β1 leads to a downregulation of miR-200b/c and E-cadherin protein expression, an upregulation of vimentin and α-SMA protein expression, an increase in the phosphorylation levels of p38 and Smad3. Pretreatment with miR-200b/c or SB203580 (a p38 inhibitor)/SIS3 (a Smad3 inhibitor) can reverse this situation (Cao et al., 2018), indicating that miR-200 may be involved in the p38 MAPK and TGF-β/Smad3 signaling pathways. The miR-133a has been reported to form a crucial negative feedback loop with the TGF-β1/Smad pathway. Moreover, pre-treatment of HFL cells with SB203580 reduces the expression of miR-133a induced by TGF-β1 and partially inhibits the expression of α-SMA and CTGF. However, pre-treatment with the PI3K/AKT signaling pathway inhibitor LY294002 only marginally reduces the upregulation of miR-133a (Wei et al., 2019). Therefore, this suggests that p38 MAPK plays a role in the anti-fibrotic mechanism of miR-133a, while the PI3K/AKT pathway may have limited involvement. Bioinformatics prediction and dual luciferase assay revealed that miR-448 targets ATP-binding cassette subfamily C member 3 (ABCC3). Following treatment with a miR-448 mimic in pulmonary fibroblast cells, cell proliferation decreased while the apoptosis rate increased. This situation could be reversed by the miR-448 inhibitor. It is noteworthy that the utilization of JNK inhibitor SP600125 led to comparable results as the miR-448 simulator, indicating that the miR-448 simulator facilitates apoptosis in lung fibroblasts and diminishes collagen synthesis by deactivating the JNK signaling pathway (Xu F. et al., 2020). In elderly patients with IPF, the expression of miR-30a-5p in bronchoalveolar lavage fluid (BALF) exosomes is downregulated, and it serves as a target of MAP3K7 binding protein 3 (TAB3). The overexpression of miR-30a-5p leads to reduced expression of TAB3, α-SMA, and fibronectin in A549 cells (Liu B. et al., 2018). These findings suggest that miR-30a may act to inhibit the MAPK pathway and impede TGF-β1 signaling, thereby serving as a key factor in the progression of IPF. Overexpression of miR-340-5p leads to decreased activating transcription factor 1 (ATF1) expression, thereby inhibiting fibroblast activation and proliferation by targeting ATF1. It also attenuates the p38 MAPK pathway following TGF-β1 stimulation, leading to reduced COL1A1 and fibronectin expression (Wei et al., 2020). To elucidate the functions of certain miRNAs, an expression profiling study utilizing microarrays revealed that the miR-19a-19b-20a subcluster can inhibit the activation of *ex vivo* fibroblasts induced by TGF-β1. This inhibition leads to the downregulation of the expression of pro-fibrotic genes such as ACTA2, COL1A1, CTGF, and Serpine1, while upregulating the expression of anti-fibrotic genes such as DCN, IGFBP5, and MMP3 (Souma et al., 2018). The researchers utilized algorithms such as TargetScan to forecast the binding of miR-19a, miR-19b, and miR-26b to the 3′UTR of CTGF, which was subsequently confirmed through dual luciferase assays. Overexpression of these miRNAs led to decreased expression of CTGF, α-SMA, and vimentin in WI-38 cells. Following treatment with ERK inhibitors PD, JNK inhibitors SP, and p38 inhibitors SB, levels of miR-19a, miR-19b, and miR-26b were elevated in WI-38 cells while the quantity of CTGF was reduced (Chen et al., 2016). This study presents initial evidence for a reciprocal interaction between MAPK activation and diminished expression of miR-19a, miR-19b, and miR-26b; suggesting that activating these miRNAs or inhibiting the MAPK pathway may ameliorate IPF (Table 3).

**TABLE 3:** The miRNAs involved in the regulation of the MAPK pathway in IPF.

MiRNA	Expression level in IPF	Experimental model	Validated target	Effect	Signal pathway	Function	Reference
miR-133a	Upregulated	Cell: HFL		Profibrotic	p38 MAPK		Wei et al. (2019)
miR-21	Upregulated	Animal: BLM fibrosis rats; Cell: MRC-5	Smad7	Profibrotic	MAPK/AP-1	Inducing the deposition of collagen	Wang et al. (2018)
miR-155	Upregulated	Animal: BLM fibrosis mice		Profibrotic	MAPK	Promote the increase of IL-4 and TGF-β	Sun et al. (2021)
miR-7219-3p	Upregulated	Animal: silica-lung fibrosis mice; Cell: RAW264.7, NIH-3T3	SPRY1	Profibrotic	Ras/ERK/MAPK	Facilitate the transdifferentiation of fibroblasts into myofibroblasts	Niu et al. (2022)
miR-552-3p	Upregulated	Sample: BALF of silicosis patients; Animal: silica-lung fibrosis mice; Cell: A549, HPAEpiC, NHLFs	CAV1	Profibrotic	MAPK	Increase expression of fibrosis markers	Li et al. (2024a)
miR-200b/c	Downregulated	Animal: The mice of early pulmonary fibrosis caused by LPS-induced ARDS; Cell: RLE-6TN	ZEB1/2	Antifibrotic	p38 MAPK, TGF-β/Smad3	Mitigate fibrosis	Cao et al. (2018)
miR-448	Downregulated	Aniaml: BLM fibrosis mice	ABCC3	Antifibrotic	JNK	Decrease the proliferation of pulmonary fibroblasts and increase the rate of apoptosis	Xu et al. (2020a)
miR-30a-5p	Downregulated	Cell: A549, BALF	TAB3	Antifibrotic	MAPK	Diminish the expression of fibrin	Liu et al. (2018a)
miR-340-5p	Downregulated	Cell: lung fibroblast	ATF1	Antifibrotic	p38 MAPK	Prevent the activation and proliferation of fibroblasts	Wei et al. (2020)
miR-19a/b,miR-26b	Downregulated	Animal: BLM fibrosis mice; Cell: WI-38	CTGF	Antifibrotic	MAPK	Inhibit fibroblast activation, downregulate expression of pro-fibrotic genes	Souma et al. (2018), Chen et al. (2016)

Abbreviations: ARDS, acute respiratory distress syndrome; ABCC3, ATP-binding cassette subfamily C member 3; BALF, bronchoalveolar lavage fluid; TAB3, MAP3K7 binding protein 3; ATF1, activating transcription factor 1.

#### 3.2.2 PI3K/AKT pathway

The PI3K/AKT pathway governs cellular growth, proliferation, metabolism, and survival. PI3K is categorized into Class I, Class II, and Class III (Engelman et al., 2006). Specifically, Class I PI3K is a heterodimer composed of a regulatory subunit p85 and a catalytic subunit p110, which can be expressed in human lung fibroblasts (Conte et al., 2011). AKT is a serine/threonine protein kinase that can be activated by upstream of PI3K. While there are three isoforms (AKT1, AKT2, and AKT3) (Revathidevi and Munirajan, 2019), researches related to pulmonary fibrosis have predominantly focused on the AKT1 and AKT2 isoforms. AKT1 plays a crucial role in positively regulating the anti-apoptotic process of alveolar macrophages (Larson-Casey et al., 2016), which is widely recognized as essential for the pathogenesis of pulmonary fibrosis. Deficiency of AKT2 can inhibit bleomycin-induced pulmonary fibrosis and inflammation (Nie et al., 2017). These findings indicate that the crucial involvement of the PI3K/AKT signaling pathway in the pathogenesis of IPF.

The available evidence indicates that overexpression of α-SMA is linked to the activation of the PI3K/AKT pathway, and the crosstalk between TGF-β and PI3K/AKT facilitates the development of pulmonary fibrosis (Sun et al., 2018). In the presence of TGF-β1, TRAF6 is capable of promoting Lys63 ubiquitination of p85, facilitating its association with TβRI. Simultaneously, p85 can also form a constitutive complex with TβRII and interact with TβRI upon exposure to TGF-β1, thereby triggering the interaction between the pleckstrin homology (PH) domain at the N-terminus of AKT and 3′-phosphatidylinositol, leading to phosphorylation at Thr308 and Ser473 residues (Hamidi et al., 2017). Downstream targets of AKT encompass mammalian target of rapamycin (mTOR), a Ser-Thr kinase that can be categorized into mTORC1 and mTORC2. Both proteins are capable of phosphorylating AKT’s Ser473 in a positive feedback loop. TGF-β1/Smad3 signaling can upregulate the expression of transcriptional activator 4 (ATF4) by activating mTORC1 and its downstream translation initiation factor 4E-binding protein 1 (4E-BP1), thereby facilitating collagen synthesis (Selvarajah et al., 2019). mTORC2 can facilitate cellular migration and invasion, contributing to the EMT induced by TGF-β1. Both of these processes may be modulated through the PI3K-Akt pathway in response to TGF-β1 activation (Bozulic and Hemmings, 2009). Hence, TGF-β1 establishes a positive feedback loop with the PI3K-Akt pathway, thereby facilitating the progression of IPF (Figure 3).

Previous research had demonstrated an upregulation of miR-21 in pulmonary fibrosis. Our establishment of a mouse model revealed that phosphatase and tensin homolog (PTEN) serves as a functional target of miR-21, and the knockout of PTEN can reverse the downregulation of EMT mediated by miR-21. Overexpression of miR-21 in pulmonary epithelial cells suppresses the expression of PTEN and enhances Akt phosphorylation (Liu Z. et al., 2019). Hence, it is evident that miR-21 may also contribute to the pathogenesis of IPF through the PTEN/Akt pathway. Astragaloside IV (AS-IV) precisely leverages this mechanism to induce autophagy and inhibit IPF, offering a promising therapeutic strategy (Li T. et al., 2024). In NIH-3T3 mouse fibroblasts treated with TGF-β1, a decrease in miR-542-5p expression was observed. Transfection of miR-542-5p mimic suppressed the proliferation and migration capacity of NIH-3T3 cells. Integrin α6 (Itgα6) is a cell surface protein associated with fibroblast proliferation and has been identified as a direct target of miR-542-5p. Overexpression of Itgα6 significantly enhances FAK/PI3K/AKT phosphorylation, promoting fibroblast proliferation and differentiation into myofibroblasts (Yuan et al., 2018). In the BLM-induced fibrosis mouse model and the lung tissue of IPF patients, miR-301a is upregulated. Deletion of the miR-301a gene in experimental mice led to reduced expression of vimentin, α-SMA, and fibronectin, thereby mitigating the severity of pulmonary fibrosis following BLM injection and suppressing the proliferation and activation of pulmonary fibroblasts (Wang J. et al., 2020). These findings suggest that miR-301a exerts negative regulation on its target gene TSC complex subunit 1 (TSC1), mediating the mTOR signaling pathway to promote structural damage in lung tissue and exacerbate the severity of pulmonary fibrosis. Upon investigation of the TGF-β1-induced EMT, it was observed that miR-483-5p expression was upregulated in human pulmonary fibrosis tissues and A549 cells. Inhibition of miR-483-5p’s target, Rho GDP dissociation inhibitor alpha (RhoGDI1), effectively counteracted the inhibiting effect of the miR-483-5p inhibitor on TGF-β1-induced EMT through the PI3K/AKT pathway (Huang et al., 2021). FOS-like 1, AP-1 transcription factor subunit (Fra-1) has been identified as a functional target of miR-34c-5p, and its expression shows a negative correlation with that of miR-34c-5p. Further investigation reveals that miR-34c-5p inhibits the PTEN/PI3K/AKT pathway. Additionally, it suppresses the proliferation and migration of human bronchial epithelial cells by interacting with PTEN/p53, thereby preventing EMT (Pang et al., 2022). A recent study on pulmonary fibrosis has revealed that the alterations in miR-423-5p and its target gene forkhead box p4 (FOXP4) have a significant impact on the expression of the PI3K/AKT/mTOR pathway (Chen et al., 2022). Utilizing mRNA and miRNA chip analysis, we identified differentially expressed genes and miRNAs in various databases, revealing the downregulation of miR-184. Subsequent validation confirmed the anti-fibrotic role of miR-184, with its expression showing a negative correlation with that of Smad2/Akt (Wang CJ. et al., 2020). Hence, miR-184 exerts an impact on pulmonary fibrosis via the TGF-β1/PI3K-AKT pathway, leading to the hypothesis that upregulation of miR-184 may mitigate fibrotic processes, thereby proposing a novel approach for managing IPF (Table 4).

**TABLE 4:** The miRNAs involved in the regulation of the PI3K/AKT pathway in IPF.

MiRNA	Expression level in IPF	Experimental model	Validated target	Effect	Signal pathway	Function	Reference
miR-21	Upregulated	Animal: irradiation mice; Cell: A549, BEAS-2B	PTEN	Profibrotic	PTEN/Akt	Induce pulmonary fibroblast activation and collagen deposition, promote TGF-β1 activity	Liu et al. (2019a), Li et al. (2024b)
miR-301a	Upregulated	Animal: BLM fibrosis mice; Cell: HFL1	TSC1	Profibrotic	mTOR	Promote the formation of EMT	Wang et al. (2020c)
miR-483-5p	Upregulated	Sample: human pulmonary fibrotic tissue; Cell: A549	RhoGDI1	Profibrotic	PI3K/AKT	Promote the formation of EMT	Huang et al. (2021)
miR-423-5p	Upregulated	Cell: BEAS-2B	FOXP4	Profibrotic	PI3K/AKT/mTOR	Promote the formation of EMT	Chen et al. (2022)
miR-542-5p	Downregulated	Animal: silica-lung fibrosis mice; Cell: NIH-3T3	Itgα6	Antifibrotic	FAK/PI3K/AKT	Inhibit proliferation and migration capabilities	Yuan et al. (2018)
miR-184	Downregulated	Cell: A549		Antifibrotic	TGF-β1/Smad, PI3K-AKT	Inhibit the TGF-β1/PI3K-AKT pathway	Wang et al. (2020a)
miR-34c-5p	Downregulated	Cell: HBE	Fra-1	Antifibrotic	PTEN/PI3K/AKT	Inhibit cell proliferation and migration, prevent EMT	Pang et al. (2022)

Abbreviations: PTEN, phosphatase and tensin homolog; RhoGDI1, Rho GDP dissociation inhibitor alpha; FOXP4, forkhead box p4; Itgα6, Integrin α6; Fra-1, FOS like 1, AP-1 transcription factor subunit; TSC1, TSC complex subunit 1.

### 3.3 The role of microRNAs in modulating supplementary fibrotic factors

In clinical and experimental mouse tissue samples of lung fibrosis, as well as in exosomes of microvascular endothelial cells, the expression level of miR-107 is significantly reduced. The anti-fibrotic activity of miR-107 is mediated through the inhibition of the HIF-1α/Notch1/PDGFRβ signaling pathway. Indeed, miR-107 directly targets the mRNA of HIF-1α, which in turn directly activates the transcription of Notch1 and PDGFRβ, thereby inhibiting ECM deposition (Wang YC. et al., 2021).

In patients with IPF, AT II cells lose their capacity to transdifferentiate into AT I cells and undergo cellular senescence and EMT. However, several studies have demonstrated that the delivery of exogenous miR-200c-3p to AT II cells enhances their transdifferentiation capacity, thereby supporting the therapeutic potential of miR-200c in IPF (Moimas et al., 2019). Subsequent investigations have identified vascular endothelial growth factor receptor 1 (Flt1) as the primary functional target of miR-200c in endothelial cells, and Flt1 depletion can effectively replicate the anti-fibrotic effects of miR-200c on pulmonary fibrosis (Volpe et al., 2023). Consequently, pulmonary endothelial cells may serve as a pertinent therapeutic target in the fight against pulmonary fibrosis.

As a competitive endogenous RNA of lncRNA PFAL, miR-18a is significantly downregulated in patients with IPF as well as in both *in vivo* and *in vitro* studies. Moreover, enhancing the expression of miR-18a through direct targeting of CTGF can mitigate TGF-β1-induced pulmonary fibrosis (Li et al., 2018).

The study by Pandit et al. (2010) demonstrated that miR-155 expression is upregulated in lung tissue from patients with IPF. This finding was further corroborated by Pottier et al. (2009). Moreover, within the downregulated transcriptome cohort, the predicted targets of miR-155 exhibited considerable diversity. A notable target is keratinocyte growth factor (KGF, FGF-7), which contains two potential binding sites for miR-155 within its 3′UTR. Both fluorescence luciferase assays and functional *in vitro* experiments have confirmed the targeting relationship between these entities. *In vivo* studies utilizing a mouse model of pulmonary fibrosis demonstrated that the expression level of miR-155 correlates with the severity of pulmonary fibrosis. Both miR-155 and the miR-17–92 cluster appear to play crucial roles in mediating the expression of soluble growth factors, including KGF and CTGF. This clearly demonstrates that a single miRNA can modulate numerous mRNA targets, while a single gene may be influenced by multiple miRNAs, resulting in synergistic effects and significant biological outcomes (Table 5).

**TABLE 5:** The miRNAs that regulate other fibrosis factors.

MiRNA	Expression level in IPF	Experimental model	Validated target	Effect	Function	Reference
miR-155	Upregulated	Animal: BLM fibrosis mice; Cell: HFL1, A549, NIH 3T3	FGF-7	Profibrotic	Stimulate cell migration	Pottier et al. (2009)
miR-145-5p	Upregulated	Sample: IPF tissues; Animal: silica-lung fibrosis mice, miR-145−/− mice; Cell: MRC-5	KLF4	Profibrotic	Regulate the differentiation of myofibroblasts	Yang et al. (2013), Sun et al. (2023)
miR-125a-5p	Upregulated	Animal: BLM fibrosis rats; Cell: IMR-90	KLF13	Profibrotic	Target KLF13 induces fibrosis	Qiu et al. (2022)
miR-142-5p	Upregulated	Cell: primary human fibroblasts, primary mouse fibroblasts	SOCS1	Profibrotic	Enhance the expression of fibrosis-promoting genes	Su et al. (2015)
miR-107	Downregulated	Sample: IPF tissues; Animal: BLM fibrosis mice; Cell: primary pulmonary microvascular endothelial cells, pericytes, epithelial cells	PDGFR β	Antifibrotic	Inhibit ECM deposition	Wang et al. (2021a)
miR-200c	Downregulated	Animal: BLM fibrosis mice; Cell: mice endothelial cells and AEC Ⅱ, human endothelial cells	Flt1	Antifibrotic	Enhance the ability of AT II cells to differentiate into AT I cells	Moimas et al. (2019), Volpe et al. (2023)
miR-18a	Downregulated	Animal: BLM fibrosis mice; Cell: mice fibroblast cells	CTGF	Antifibrotic	Reduce pulmonary fibrosis and EMT	Li et al. (2018)
miR-133a	Downregulated	Animal: BLM fibrosis mice; Cell: HFL, 16HBE, NIH3T3	CTGF	Antifibrotic	Resist fibrosis through the p38 MAPK pathway	Wei et al. (2019)
miR-19a/b, miR-26b	Downregulated	Animal: BLM fibrosis mice; Cell: WI-38	CTGF	Antifibrotic	Inhibit fibroblast activation, downregulate expression of pro-fibrotic genes	Souma et al. (2018), Chen et al. (2016)

Abbreviations: FGF-7, keratinocyte growth factor; KLF4, Krüppel-like factor 4; SOCS1, Suppressor of Cytokine Signaling 1; Flt1, vascular endothelial growth factor receptor 1.

## 4 The utilization of miRNAs in therapeutic applications

Although nintedanib and pirfenidone are recommended by guidelines for the treatment of IPF (Raghu et al., 2022), neither of these drugs can fully halt the progression of IPF, and they are associated with adverse events such as gastrointestinal side effects, photosensitive rashes, and elevated liver function levels (Richeldi et al., 2014; Noble et al., 2016). Lung transplantation can prolong the survival period of IPF patients; however, only 66% of them survive beyond 3 years post-transplantation, and merely 53% survive beyond 5 years (Valapour et al., 2018). Consequently, the treatment options for IPF remain limited, and there is still an unmet need for new anti-fibrotic therapies. Given their role as signaling pathways and cell function regulators, miRNAs may offer a promising alternative approach for addressing IPF at present.

The primary objective of miRNA therapy is to effectively alter and reverse the expression changes associated with pathological miRNAs. This encompasses reducing the expression of pathogenic miRNAs that contribute to disease progression or inhibiting their function, as well as enhancing or restoring the pathological inhibitory effects of endogenous miRNAs. Designing miRNA-targeted therapy trials at the gene level presents an intriguing research perspective. At present, miRNA levels are commonly modified through the use of nucleic acid molecules, which includes synthetic double-stranded miRNAs, recombinant expression vectors that encode miRNA sequences, and oligonucleotide-based miRNA inhibitors (van Rooij and Kauppinen, 2014). Krützfeldt et al. (2005) were the first to utilize antagomir for *in vivo* miRNA silencing. Additionally, systemic administration of miR-29 mimic MRG-201 has been shown to reduce fibrosis in animal models (Montgomery et al., 2014). In comparison to double-stranded miR-29 mimics, the “miR-29b Psh-match” developed by Yamada et al. features the single-stranded RNA structure and demonstrates more potent therapeutic effects in mice induced by BLM, making it better suited for the treatment of IPF patients (Yamada et al., 2017). Based on MRG-201, a novel miR-29 mimic, MRG-229, was developed with additional sugar modifications and conjugation with the internalization molecule BiPPB (PDGFβR-specific bicyclic peptide). This new generation of miRNA can effectively reduce the expression of pro-fibrotic genes and collagen production in fibrotic cells, animals, and humans, as well as has good tolerability at clinically relevant doses (Chioccioli et al., 2022). Artificially engineered miRNA constructs, referred to as “amiRNAs,” are composed of siRNA sequences combined with pri-miRNA transcriptional scaffolds, yielding a high degree of target specificity (Kotowska-Zimmer et al., 2021). AMT-130 is an amiRNA-based therapeutic agent composed of siRNA sequences that specifically target the Huntington gene, along with a pri-miR-451 scaffold. It is currently undergoing clinical trials for the treatment of this condition (NCT04124093) (Keskin et al., 2019). The discovery of this innovative miRNA may represent a significant advancement in the *in vivo* treatment of IPF. As further research uncovers the mechanisms of action of miRNA, utilizing *in vivo* injection of miRNA mimics or antagonists, and using miRNA alternative therapies to investigate their therapeutic effects of miRNAs in the body will elevate our understanding and application of miRNA to a new level.

While the therapeutic potential of miRNA is undeniably compelling, to date, only a limited number of miRNA-based drugs have progressed to clinical trial stages. Miravisen (Roche) functions as an antimiR targeting miR-122 for the treatment of hepatitis C virus (HCV) infection (NCT01200120) (Janssen et al., 2013). Remlarsen, a chemically modified naked miR-29b mimic, exerts its effects by inhibiting scar formation through anti-fibrotic mechanisms (NCT02603224, NCT03601052) (Gallant-Behm et al., 2019). TargomiRs is a tumor suppressor that encapsulates a miR-16 mimic within non-viable bacterial microcells, specifically targeting the epidermal growth factor receptor (EGFR) (NCT02369198) (MacDiarmid et al., 2007). MRX34 is a miR-34a mimic encapsulated in lipid nanoparticles, which was ultimately discontinued in 2016 due to immune-related serious adverse events (NCT01829971) (Hong et al., 2020). Nevertheless, the expression and function of miRNAs in IPF had been investigated in clinical trials dating back to 2010 (NCT00258544) (Pandit et al., 2010). In the comparison of 10 control tissues and 10 IPF tissues, let-7d exhibited a significant reduction in IPF lungs, and the number of epithelial cells expressing let-7d showed a positive correlation with lung function. *In vivo*, suppression of let-7d led to thickening of the alveolar septum. However, there have been no additional clinical trials to assess the impact of targeting specific miRNAs on IPF. In animal models of lung disease, significant efficacy has been demonstrated following tracheal injection administration. Subsequently, clinical research regarding the application of miRNAs in IPF experienced a period of stagnation. In a systematic review (Bagnato et al., 2017), miR-29a/b/c-3p, miR-21-5p, miR-92a-3p, miR-26a-5p and let-7d-5p were identified as having a distinct modulatory effect associated with pulmonary fibrosis in IPF. Subsequently, the first open-label trial conducted in humans (NCT02874989) (Justice et al., 2019) provided evidence that the combination of dasatinib and quercetin for treating IPF was associated with changes in bodily functions that correlate with alterations in miRNAs, thereby affirming the role of miRNAs in this context and warranting further evaluation in larger-scale randomized controlled trials. Given that a subset of post-COVID-19 patients may develop progressive pulmonary fibrosis, it is hypothesized that miRNAs could serve a similar role in both conditions (Guiot et al., 2023). It is unequivocal that the dysregulation of fibrosis-related miRNAs, characterized by elevated levels of miR-21-5p and reduced levels of miR-141-3p, may contribute to the progression of pulmonary fibrosis in patients recovering from COVID-19. In a separate clinical study, the integration of transcriptomic and proteomic data revealed that miRNAs can further stratify IPF patients based on heatmap visualization and differential expression analysis (NCT01915511) (Ruan et al., 2023). This approach may facilitate the identification of individuals at risk for disease progression and enhance clinical trial design.

Although the potential of miRNA as a therapeutic drug awaits validation through clinical trials, current research is predominantly focused on the therapeutic properties of exosomal miRNA derived from mesenchymal stem cells (MSCs). MSCs possess robust anti-inflammatory, anti-fibrotic, and immunomodulatory capabilities, positioning them as a novel avenue for non-cellular therapy in IPF (Cahill et al., 2016; Glassberg et al., 2017; Mansouri et al., 2019). Hydrogen sulfide has been incorporated into the medical field for its anti-fibrotic properties and enhanced MSC function. Treatment with NaHS and bone mesenchymal stem cells (BMSCs) can attenuate pulmonary fibrosis, downregulate miR-21, and upregulate lncRNA-GAS5 expression, leading to improved lung function, reduced inflammation, and fibrosis markers (Nassar et al., 2022). Similarly, studies have demonstrated that dysregulation of miR-29 and miR-199 induced by BLM is restored after adipose-derived mesenchymal stem cells (ASCs) treatment (Periera-Simon et al., 2021), which may be partly attributed to the downregulation of miR-199 and its corresponding target caveolin-1 and phosphorylated PKB protein (Rubio et al., 2018). Additionally, ASC-EVs can facilitate the regression of pulmonary fibrosis through the intercellular transfer of miR-29c and miR-129 (Nataliya et al., 2023). Recent research has shown that human umbilical cord mesenchymal stem cell-derived extracellular vesicles (HUMSC-EVs) as a carrier can deliver miR-223-3p to inhibit circPWWP2A, thereby alleviating pulmonary fibrosis through the NLRP3 signaling pathway (Hou et al., 2023). Similarly, miR-218 from HUMSC-EVs reduces EndMT, decreases CpG island methylation at the BMP2 promoter, and increases BMP2 (Zhao et al., 2023). Yi Lu et al. discovered that inhibition of m6A RNA methylation prevents LR-MSCs from differentiating into myofibroblasts by METTL3 (methyltransferase 3)/miR-21/PTEN signaling pathway (Lu et al., 2023) (Table 6).

**TABLE 6:** The miRNAs that have the potential to be utilized as therapeutic agents in IPF.

Origin	Exsomal miRNAs	References
BMSCs	miR-21-5p, miR-22-3p, miR-34a-5p, miR-148a-3p, miR-196a-5p, miR-199a/b-3p, miR-199b-5p, miR-630, miR-29b-3p, miR-186, miR-30b	Nassar et al. (2022), Shentu et al. (2017), Wan et al. (2020), Zhou et al. (2021), Zhu et al. (2024b)
HP-MSCs	miR-214-3p	Lei et al. (2021)
ASC	miR-29, miR-199, miR-129	Periera-Simon et al. (2021), Nataliya et al. (2023), Shi et al. (2021a)
HUMSC	miR-21, miR-23, miR-148a-3p, miR-223-3p, miR-218	Zhao et al. (2023), Hou et al. (2023), Shi et al. (2021b), Jiang et al. (2023)
LR-MSC	miR-7a-5p, miR-140-3p, miR-148-3p, miR-152-3p, miR-27a/b-3p, miR-34a/c-5p, miR-128-3p, miR-224-5p, miR-323-3p, miR-21	Lu et al. (2023), Wang et al. (2020d)
Pulmonary epithelial cells	miR-34b, miR-142-3p, miR-144-3p, miR-503-5p, miR-223, miR-27b-3p	Parimon et al. (2019), Feng et al. (2021)
Vascular endothelial cells	miR-223, miR-27b-3p, let-7d	Xie et al. (2020), Feng et al. (2021)
MenSCs	let-7	Sun et al. (2019), Sun et al. (2024)
Macrophages	miR-142-3p, miR-125a-5p	Guiot et al. (2020), Ding et al. (2023)
LSCs	miR-99a-5p, miR-100-5p, miR-30a-3p, let-7	Dinh et al. (2020)
Adipose	miR-122-5p	Qi et al. (2022)
HBECs	miR-16, miR-26a, miR-26b, miR-141, miR-148a, miR-200a	Kadota et al. (2021)

Abbreviations: BMSC, bone marrow mesenchymal stem cell; HP-MSC, human placenta-derived mesenchymal stem cell; ASC, adipose-derived mesenchymal stem cell; HUMSC, human umbilical cord mesenchymal stem cell; LR-MSC, lung resident mesenchymal stem cell; MenSC, menstrual blood stem cell; LSC, lung spherocyte; HBEC, human bronchial epithelial cell.

In recent years, there has been continuous advancement in the development of natural products, with small molecule compounds being utilized as alternative therapies for pulmonary fibrosis through the regulation of various signaling pathways (Li and Kan, 2017). Paclitaxel (PTX), as a diterpenoid alkaloid exhibiting anti-cancer properties, it has the ability to reverse AEC’s EMT, enhance miR-140 expression, suppress Smad3 and p-Smad3, and elevate E-cadherin levels. Notably, miR-140 can also facilitate the low-dose PTX-induced anti-pulmonary fibrosis effect (Wang et al., 2013). Resveratrol (Res) can counteract the upregulation of miR-21 induced by BLM by reducing TGF-β1 and p-Smad2/3 levels, as well as inhibiting the phosphorylation of ERK, JNK, and p38, thereby ameliorating IPF (Wang et al., 2018). Ligustrazine inhibits the PI3K/Akt/mTOR and Hedgehog signaling pathways through upregulation of miR-193a expression and induction of autophagy, concomitant with downregulation of TGF-β1, CTGF, collagen I and III expression (Liu MW. et al., 2019). Astragalus is a promising natural medicinal product, and one of its main bioactive components, Astragaloside IV (ASV), has been shown to induce autophagy in IPF models by inhibiting the expression of miR-21, thereby repressing the activation of the PTEN/PI3K/AKT/mTOR pathway (Li T. et al., 2024). Meanwhile, ASV downregulates the expression of lncRNA-ATB and upregulates miR-200c, leading to the suppression of ZEB1 and inhibition of the EMT process (Guan et al., 2024).

## 5 Anticipated future challenges and recapitulation

Research on miRNA has yielded novel insights into the pathogenesis of IPF, elucidating new disease progression pathways and potentially efficacious therapeutic interventions. Although our understanding of IPF development is rapidly advancing, further research efforts are necessary to complement and enhance our comprehension. The scientific validity and clinical applicability of miRNA as a crucial factor in IPF need to be scrutinized in terms of the following issues.

A single miRNA has the capacity to regulate numerous mRNA targets; conversely, a single gene may be modulated by multiple miRNAs, resulting in synergistic effects and significant biological outcomes. While this characteristic positions miRNA as a potentially powerful therapeutic agent, it also poses challenges in managing adverse reactions observed during clinical trials. One of the significant challenges is the off-target effects associated with miRNAs, which are detrimental and phenotype-related; however, they are often overlooked due to the difficulties in assessment and control. Off-target effects refer to the unintended specific silencing of non-target mRNAs by miRNA molecules resulting from inadvertent hybridization (Jackson et al., 2003), thereby complicating the overall response. This pattern is not influenced by the concentration of molecules or cell type, in a sense indicating that it exhibits specific effects. When exogenous miRNAs are overexpressed in the body, they compete with endogenous miRNAs for a limited pool of Exp5 (Jackson and Linsley, 2010), Ago, and RISC (Seok et al., 2016), thereby saturating the miRNA mechanisms necessary for normal endogenous functions. As a component of the off-target effect, this saturation frequently results in cytotoxicity or global disruption of gene expression (Jacquet et al., 2021). However, there is currently a paucity of data quantitatively describing the dose-dependent regulation of target genes by miRNAs, necessitating further quantitative investigations under both physiological and pathological conditions. In contrast to siRNA interference, miRNA-mediated functions are regulated by multiple layers (Wang P. et al., 2021). The understanding of potential regulatory mechanisms—including IsomiRs, RNA-binding proteins (RBPs), alternative polyadenylation (APA), ceRNA, and miRNA modifications—remains limited. This intricate regulatory network presents challenges for the application of miRNAs but may also confer certain advantages. In comparison to shRNA, the interest in amiRNA has been comparatively diminished, potentially due to the increased complexity of amiRNA design, which results in lower predictability of outcomes and consequently reduced silencing efficiency. Moreover, in the majority of studies, both the specificity of amiRNA treatment and the levels of amiRNA expression have not been evaluated (Kotowska-Zimmer et al., 2021). Furthermore, the expression of endogenous miRNA-3p and -5p strands is not regulated, which renders exogenous miRNAs produced by DNA/viral vectors or synthetic pre-miRNAs insufficient for providing definitive therapeutic applications (Zhou et al., 2016; Schober et al., 2014). Simultaneously, the immune response induced by dsRNA, including the activation of Dicer-associated antiviral pathways, necessitates the use of siRNA for effective gene silencing in mammalian cells (Karpala et al., 2005). Furthermore, the exosomes previously mentioned represent a highly versatile delivery system; however, they carry the risk of inducing immune responses, and the challenges associated with endosomal escape following internalization are also concerning.

The current data on miRNA gene expression in pulmonary fibrosis is mostly collected from mixed cell tissues of the entire lung and primary cells isolated from human tissues are known for their sample heterogeneity, including fibrotic and non-fibrotic, severely fibrotic and diffusely fibrotic areas of the same lobe of the lung. Additionally, using isolated and cultured fibroblasts may mask the epigenetic changes in the microenvironment that occur within fibrotic foci within the body. These factors related to cell type heterogeneity and *in vitro* culture environment (Sabater et al., 2023) may drive the lack of overlap between miRNA expression and expected gene expression. One potential solution to this issue could involve decomposing whole-tissue expression into cell-specific components and utilizing laser capture microdissection (LCMD) methods. The latter can isolate fibroblasts or epithelial cells from the specific anatomical regions of resected lung tissue, thereby reducing sample heterogeneity and enhancing our understanding of the role of miRNA in IPF pathogenesis.

A significant challenge in targeting miRNAs for IPF is the uncertainty regarding whether the genes regulated by a single miRNA differ between mice and humans. The variability in study sample sources—such as different cell lines and fibrosis models in either humans or mice—complicates the ability to correlate the distinct expression levels and effects of each individual miRNA with the clinical realities faced by IPF patients. The absence of miR-145 in mice results in enhanced ECM production by vascular smooth muscle cells (Zhao et al., 2015), while simultaneously conferring protection against BLM-induced pulmonary fibrosis (Yang et al., 2013). The contradictory findings not only emphasize the heterogeneity of the tissue but also highlight that information derived from murine models remains limited; while it aids in elucidating general mechanisms of fibrosis, it remains uncertain whether these mechanisms are applicable to human diseases. Moreover, the classic BLM-induced mouse model of pulmonary fibrosis fails to fully replicate IPF, primarily due to the rapid onset of symptoms and the partial reversibility of the fibrotic process. Furthermore, sequence variations in the 3′UTR regions across different species diminish the likelihood of accurately predicting off-target effects in humans based on mouse models. Consequently, future research should not only focus on the continuous refinement of existing models but also emphasize the testing and validation of hypotheses across multiple model systems.

The utilization of miRNA-containing EVs for diagnosis and therapy in IPF is undeniably a feasible avenue. However, given the diverse origins of EVs from various bodily fluids and their specificity to organs, tissues, and cells, precise identification and potency testing are imperative, including the classification in conjunction with traditional biomarkers. For example, the content of miRNA in serum EVs exhibits a strong correlation with CD9 positivity levels, which can be standardized. Furthermore, our focus lies on the net change in miRNA content within EVs, rather than the total quantity present in the sample, as it reflects cellular or tissue conditions during disease progression. For these reasons, the isolation and purification technologies for EVs also require high heterogeneity, and urgently need a comprehensive system of standards for manufacturing processes and regulatory requirements to ensure quality control and reproducibility. This will facilitate the broader application of specific cell-originated EVs in IPF treatment trials. A recent report introduced a method for culturing HUMSC-EVs in microcarrier suspensions using a 3D dynamic system (Xu C. et al., 2020), which can be used for large-scale manufacturing in clinical practice. In addition, factors such as the number of cell passages, seeding density (Patel et al., 2017), and oxygen content (Teixeira et al., 2015) during culture, as well as the stability and integrity of MSC-Exos during storage and transportation will also impact the composition and yield of EVs. Additionally, the functionality of MSCs diminishes with age and disease conditions, resulting in alterations in their potential for multipotent differentiation and the emergence of aging phenotypes in patients with abdominal aortic aneurysm (Huang X. et al., 2019), diabetes (Capilla-González et al., 2018), and lung injury (Huang R. et al., 2019), ultimately leading to minimal therapeutic effects. Considering that IPF is an age-related disease, MSCs from IPF patients (IPF-MSCs) may exhibit reduced beneficial effects on IPF. Recently, strategies such as genetic modification and supplementation with cytokines have been employed to rejuvenate aging MSCs and augment their therapeutic benefits (Liu et al., 2020). A novel intervention strategy aimed at restoring the vigor of aging MSCs by regulating key miRNAs like miR-199 (Shi et al., 2021a) presents a promising new candidate target for enhancing the therapeutic efficacy of MSCs in addressing lung fibrosis-related diseases.

In conclusion, previous research has highlighted the pivotal role of miRNA in the pathophysiology of IPF. It not only modulates the state of mRNA to change the expression of target genes but also dynamically regulates DNA methylation, and other ncRNAs, including itself. It's like turning on a signal indicator, these regulatory mechanisms lead to changes in miRNA, which generate a cascading amplification reaction in the TGF-β1/Smad, MAPK, and PI3K/AKT pathways, ultimately resulting in the expression of fibrotic phenotypes. The design of miRNA at the genetic level represents a promising therapeutic strategy, wherein miRNA-based therapeutics are integrated into both *in-vivo* and *in-vitro* applications, potentially resulting in the most immediate clinical outcomes. In addition, utilizing EVs, particularly MSCs, as a delivery system for miRNA has demonstrated promising therapeutic efficacy in the treatment of IPF. Further research into the role of miRNA in natural compounds is also warranted. Nevertheless, the pivotal role of miRNA within regulatory networks and its diverse effects are regarded as significant challenges confronting miRNA-based therapeutic approaches. A thorough and systematic functional characterization of individual candidate miRNAs is essential for their therapeutic application. MiRNAs may represent a new level of treatment for IPF disease, and it is an important research question to update our understanding of how these miRNAs intervene in the development of IPF.
